# ECD1 functions as an RNA-editing *trans*-factor of *rps14*-149 in plastids and is required for early chloroplast development in seedlings

**DOI:** 10.1093/jxb/ery139

**Published:** 2018-04-10

**Authors:** Tian Jiang, Jing Zhang, Liwei Rong, Yanjiang Feng, Qi Wang, Qiulai Song, Lixin Zhang, Min Ouyang

**Affiliations:** 1 Photosynthesis Research Center, Key Laboratory of Photobiology, Institute of Botany, Chinese Academy of Sciences, Beijing, China; 2 University of Chinese Academy of Sciences, Beijing, China; 3 Cultivation and Crop Tillage Institute of Heilongjiang Academy of Agricultural Sciences, Harbin, China

**Keywords:** Arabidopsis, chloroplast development, cotyledon, Early Chloroplast Development 1 (ECD1), early stages, pentatricopeptide repeat protein (PPR), RNA editing

## Abstract

Chloroplast development is a highly complex process and the regulatory mechanisms have not yet been fully characterized. In this study, we identified Early Chloroplast Development 1 (ECD1), a chloroplast-localized pentatricopeptide repeat protein (PPR) belonging to the PLS subfamily. Inactivation of *ECD1* in Arabidopsis led to embryo lethality, and abnormal embryogenesis occurred in *ecd1*/+ heterozygous plants. A decrease in *ECD1* expression induced by RNAi resulted in seedlings with albino cotyledons but normal true leaves. The aberrant morphology and under-developed thylakoid membrane system in cotyledons of RNAi seedlings suggests a role of *ECD1* specifically in chloroplast development in seedlings. In cotyledons of *ECD1*-RNAi plants, RNA-editing of *rps14*-149 (encoding ribosomal protein S14) was seriously impaired. In addition, dramatically decreased plastid-encoded RNA polymerase-dependent gene expression and abnormal chloroplast rRNA processing were also observed. Taken together, our results indicate that *ECD1* is indispensable for chloroplast development at the seedling stage in Arabidopsis.

## Teaser

The pentatricopeptide repeat protein ECD1 functions as an RNA-editing *trans*-factor in plastids of cotyledons and is necessary for early chloroplast development in Arabidopsis seedlings.

## Introduction

Chloroplasts are not only the exclusive organelles that perform photosynthesis but they are also responsible for many other biosynthetic processes, such as the synthesis of amino acids, hormones, and metabolites (Sakamoto *et al.*, 2008). The development of functional chloroplasts is a prerequisite for photosynthesis and is also tightly co-ordinated with plant growth and development. Chloroplast gene expression is crucial for chloroplast development, and is carried out by two kinds of RNA polymerases: plastid-encoded bacterial-type RNA polymerase (PEP) and nuclear-encoded phage-type RNA polymerase (NEP) (Hedtke *et al.*, 1997; Liere and Maliga, 2001; Börner *et al.*, 2015). Given their increasing activity during chloroplast development, PEPs are clearly crucial for chloroplast development at early stages of plant growth (Mullet, 1993). During chloroplast biogenesis, plastid ribosomal proteins are required to establish a functional chloroplast translational apparatus and deficiency of these proteins leads to lethality (Tiller and Bock, 2014). The successful assembly of ribosomal proteins in chloroplast is therefore very important for chloroplast development.

 Derived through endosymbiosis from cyanobacteria, chloroplasts are semi-autonomous organelles that have their own genome. However, there are only about 100–150 genes in the plastid genome, the products of which are mainly involved in photosynthesis and plastid gene expression (Sato *et al.*, 1999). The vast majority of chloroplast proteins (>2000) are encoded in the nucleus, translated in the cytosol, and then imported into the chloroplast. Thus, the formation of functional chloroplasts relies on co-ordination of gene expression between the plastid and the nucleus. Chloroplast gene expression is regulated by a set of nuclear-encoded factors. Among these, pentatricopeptide repeat (PPR) proteins, which constitute one of the largest protein family in land plants, have been demonstrated to play important roles in chloroplast gene expression and function. Mutations of PPR genes usually result in seedling-lethal or embryo-lethal phenotypes. The Arabidopsis genome encodes more than 450 members of this family (Lurin *et al.*, 2004; Shikanai and Fujii, 2013), and almost all of them are predicated to localize to plastids or mitochondria (Lurin *et al.*, 2004). Members of the family are characterized by the PPR motif, which appear as tandem repeats of a highly degenerate unit of 35 amino acids (Small and Peeters, 2000; Lurin *et al.*, 2004). The PPR protein family is classified into P and PLS subfamilies (Lurin *et al.*, 2004), the latter being specific to land plants. The P subfamily usually does not contain any other conserved motifs except for the canonical PPR (P) motifs. By contrast, the PLS subfamily contains long (L) and short (S) PPR-like motifs as well as classic PPR motifs. In addition, based on the presence of different C-terminal motifs, the PLS subfamily is further divided into the PLS, E, E+, and DYW subgroups (Schmitz-Linneweber and Small, 2008).

PPR proteins have been reported to be involved in almost all stages of chloroplast gene expression. For example, PPR10 is required for the accumulation of processed RNAs with the 5′ or 3′ terminus in the *atpI-atpH* or *psaf-rpl33* intercistronic region (Pfalz *et al.*, 2009; Barkan and Small, 2014). CRR2 was the first reported DYW-PPR protein and it is involved in the intergenic RNA cleavage between *rps7* and *ndhB* (Hashimoto *et al.*, 2003; Shikanai and Fujii, 2013). There are *ppr* mutants that affect PEP-dependent gene expression, such as *dg1* (Chi *et al.*, 2008). SOT1, a PPR protein with a small MutS-related (SMR) domain has endonuclease activity. Its PPR domain specifically recognizes a 13-nucleotide RNA sequence in the 5′ end of the chloroplast 23S-4.5S rRNA precursor (Zhou *et al.*, 2017). A rice mutant, *wsl*, in which a PPR protein WSL is missing, exhibits reduced translation efficiency caused by abnormal splicing of the *rpl2* gene (Wang *et al.*, 2017). The PPR protein EMB2654 is required for the *trans*-splicing of the plastid *rps12* transcript and its binding site is localized on one of the intron halves (Aryamanesh *et al.*, 2017).

Despite the presence of numerous PPR proteins in higher plants, their functions in the regulation of chloroplast development has not yet been elucidated. Here, we report a novel chloroplast factor, Early Chloroplast Development 1 (ECD1), that is involved in early chloroplast development in seedlings and which belongs to the PLS subgroup of the PPR family. Disruption of *ECD1* leads to embryo lethality and RNAi lines display albino cotyledons but normal leaves. Aberrant chloroplast ultrastructure and deficient RNA-editing of *rps14*-149 (encoding ribosomal protein S14) in plastids were detected in cotyledons of the *ECD1*-RNAi transgenic plants. Mutation of *ECD1* also resulted in decreased expression of PEP-dependent genes and abnormal rRNA processing. Our results indicate that *ECD1* plays a vital role in chloroplast development in seedlings.

## Materials and methods

### Plant materials and growth conditions

We obtained the T-DNA insertion line CS16045 (ecotype Columbia) from the Arabidopsis Biological Resource Center (https://abrc.osu.edu/; last accessed 27 April 2018). This line is the same as the one used in previous studies by Tzafrir *et al.* (2004) and Cushing *et al.* (2005). The T-DNA insertion was confirmed by PCR with T-DNA-specific primers. Seeds of wild-type and mutant plants were surface-sterilized after incubation at 4 °C for 3 d to synchronize germination, then sown on Murashige and Skoog (MS) medium containing 2% (w/v) sucrose. Plants were grown in soil under a 12/12-h light/dark cycle with a photon flux density of 120 μmol m^–2^ s^–1^ at 22 °C. For lincomycin and spectinomycin treatment, the wild-type seeds were surface-sterilized and placed on MS media with the addition of 500 μM lincomycin or 50 mg l^–1^ spectinomycin. The seedlings were harvested after 7 d.

To produce *ECD1* knock-down plants, an RNAi construct for *ECD1* was generated. A fragment of 434 bp of the *ECD1* gene (from nucleotides 286 to 719) was amplified and inserted into the PFGC5941 vector. The forward restriction endonucleases were *NcoI* and *SwaI*, and the reverse enzymes were *XbaI* and *BamHI*. The constructs were transformed into *Agrobacterium tumefaciens* strain GV3101 and introduced into the wild-type plants by the floral dip method (Clough and Bent, 1998). Transgenic plants were selected on MS medium containing 50 μg ml^–1^ Basta.

### Subcellular localization of GFP proteins

DNA encoding the 218 N-terminal amino acids of ECD1 was amplified and ligated into the green fluorescent protein (GFP) fusion vector pUC18-35S-sGFP with GFP as a reporter. The controls with mitochondrial-, chloroplast-, and nuclear-localization signals were FROSTBITE1 (FRO1), ribulose bisphosphate carboxylase small subunit (RbcS), and PTM-N (Sun *et al.*, 2011), respectively. The resulting fusion constructs and the control vectors were introduced into Arabidopsis mesophyll protoplasts according to the PEG-mediated method (Kovtun *et al.*, 2000). Fluorescence analysis was performed on an LSM 510 Meta confocal laser scanning system (LSM510; Carl Zeiss, Jena, Germany).

### RNA gel blotting, RT-PCR, and quantitative RT-PCR

Total leaf RNA was extracted from 7-d-old seedlings, and from 14-d-old cotyledons and true leaves using an RNeasy Plant Mini kit (Qiagen). RNA concentration was determined using thermo NanoDrop 2000. Total RNA from seedlings of the wild-type and the *ECD1*-RNAi-1 line was separated on 1.3% (w/v) agarose-formaldehyde gels, blotted to a nylon membrane, and subsequently hybridized with a probe labeled with ^32^P. The probes were prepared by PCR amplification and labeled using the Prime-a-Gene Labeling System (SGMB01-Promega-U1100).

RNA was used to generate first-strand cDNA in a 20-μl reaction using the Superscript III cDNA synthesis system (Invitrogen). The resulting cDNA samples were used as templates for RT-PCR analysis. Quantitative RT-PCR was performed using the SYBR Premix ExTaq Kit (Takara) following the manufacturer’s instructions with a Light Cycler 480 system. The expression level was normalized to that of an *ACTIN* control.

### Histochemical GUS staining

Tissues were incubated in cold 90% (v/v) acetone and placed in a staining buffer (100 mM sodium phosphate buffer, pH 7.2, 0.2% Triton X-100, 10 mM potassium ferrocyanide, 10 mM potassium ferricyanide, 0.25M EDTA, and 1 mM X-gluc). After vacuum-infiltration for 15–30 min, samples were incubated for 16–24 h at 37 °C. β-Glucuronidase (GUS)-stained tissues were cleared with an ethanol series of 20% (v/v), 30% (v/v), and 50% (v/v) for 30 min in turn, and finally incubated in a solution of 70% (v/v) ethanol and 30% (v/v) acetic acid for at least 30 min until the tissues became transparent enough to observe under a dissecting Olympus SZX16 microscope.

### Protein isolation and immunoblot analysis

Total proteins were prepared as previously described (Martínez-García *et al.*, 1999). Protein concentrations were determined using the Bio-Rad DC protein assay. For immunoblot analysis, total proteins were separated by SDS-PAGE and transferred to nitrocellulose membranes. The membranes were incubated with specific primary antibodies, and the signals were detected using a Pro-Light HRP Chemiluminescent Kit (Tiangen Biotech). PsaA, D1, LHCII, Cytb6, Cytf, CF0II, and RPS14 were expressed and purified in-house at our laboratory and used to generate polyclonal antibodies in rabbits. The antisera for RPL2 and RPS3 were provided by Tiegang Lu, and the anti-FLAG antibody was obtained from Abmart (www.ab-mart.com/; last accessed 27 April 2018).

### Analysis of RNA editing

A series of specific primers were used to amplify the regions of the genes containing the editing sites in Arabidopsis (Cai *et al.*, 2009) from the cDNA using RT-PCR, and the products were sequenced directly (for a list of primers used in this study see Supplementary Table S1 at *JXB* online). The levels of RNA editing were estimated by the relative heights of the peaks of the nucleotide in the sequence analysed. Plasmids prepared from approximately 90 independent colonies of each sample were sequenced to determine the RNA-editing efficiency of *rps14*-80 and *rps14*-149.

### Transmission electron microscopy

For TEM processing, wild-type and *ECD1*-RNAi-1 leaves from 7-d-old plants, and from cotyledons and true leaves from 14-d-old plants were collected. The tissue was cut into small pieces and fixed in 3% glutaraldehyde in phosphate buffer for 4 h at 4 °C. After fixation, the tissue was rinsed in phosphate buffer 3–4 times and then post-fixed in 1% OsO_4_ overnight at 4 °C. After rinsing in phosphate buffer again, the samples were dehydrated in an ethanol series, infiltrated with a graded series of epoxy resin in epoxy propane, and embedded in Epon 812 resin. Thin sections were obtained using a diamond knife on a Reichert OM2 ultramicrotome, stained with 2% uranylacetate, pH5.0, followed by 10 mM lead citrate, pH12, and viewed with a transmission electron microscope (JEM-1230; JEOL).

### Analysis of embryo development

Embryos were excised from wild-type and *ecd1+/* siliques at different developmental stages and cleared in Hoyer’s solution (7.5 g gum arabic, 100 g chloral hydrate, and 5 ml glycerol in 30 ml water) as described by Meinke (1994). Embryo development was studied microscopically using an Olympus BH-2 microscope equipped with Nomarski optics.

### Bimolecular fluorescence complementation assays

BiFC assays were performed as previously described (Walter *et al.*, 2004). Full-length cDNA of *ECD1* was cloned into pSAT4A-nEYFP-N1, and full-length cDNAs of *MORF2* and *MORF9* (multiple organellar RNA-editing factor) were cloned into pSAT4A-cEYFP-N1. The plasmids were co-transformed into protoplasts. Yellow fluorescent protein (YFP) was imaged using a confocal laser scanning microscope (LSM510; Carl Zeiss, Jena, Germany).

### Yeast two-hybrid assays

The CDS of *ECD1* without the first 150 bp encoding the transit peptide was amplified by PCR and cloned into pGBKT7 DNA-BD as bait. Sequences encoding the mature MORF2, MORF3, MORF6, MORF8, and MORF9 proteins were cloned into pGADT7 as prey. The prey and bait constructs were co-transformed into Y2HGold yeast cells. The transformation was performed using the Matchmaker Gold Yeast Two-Hybrid System (Clontech) according to the manufacturer’s instructions. Interactions were determined by growing diploid yeast colonies on synthetic dropout (SD) medium containing 40 μg ml^–1^ X-α-Gal (5-bromo-4-chloro-3-indolyl-α-d-galactopyranoside) without tryptophan, leucine, and histidine.

### RNA immunoprecipitation assays

RIP assays were performed as described previously (Kim *et al.*, 2012) using 7-d-old *35S::ECD1-FLAG* transgenic seedlings. Anti-FLAG M2 magnetic beads were obtained from Sigma (M8823). Protein A/G Sepharose incubated with pre-immune serum was used as the control. RNA was isolated by phenol-chloroform isoamyl alcohol extraction and then analysed by qRT-PCR.

### Electrophoretic mobility shift assays

EMSAs were carried out using a LightShift Chemiluminescent RNA EMSA Kit (Thermo 20158). After incubation for 20 min at 25 °C, the samples were resolved on a 6% Tris-borate gel in 0.5× TBE buffer, transferred to a nylon membrane, and subsequently processed using a chemiluminescent detection kit (Thermo 89880). The 5′-end biotin-labeled oligoribonucleotides *rps14*-80 and *rps14*-149 were synthesized and labeled by Takara Bio Inc. To produce recombinant MBP-ECD1 proteins, the coding sequence of *ECD1* lacking the transit peptide sequence was PCR-amplified, digested with *SacI* and *NotI*, and inserted into pETMALc-H (Pryor and Leiting, 1997). Recombinant protein was expressed and then purified by amylose affinity chromatography according to the manufacturer’s instructions (New England BioLabs).

## Results

### Mutations in *ECD1* produce defects in embryogenesis

**Fig. 1.**
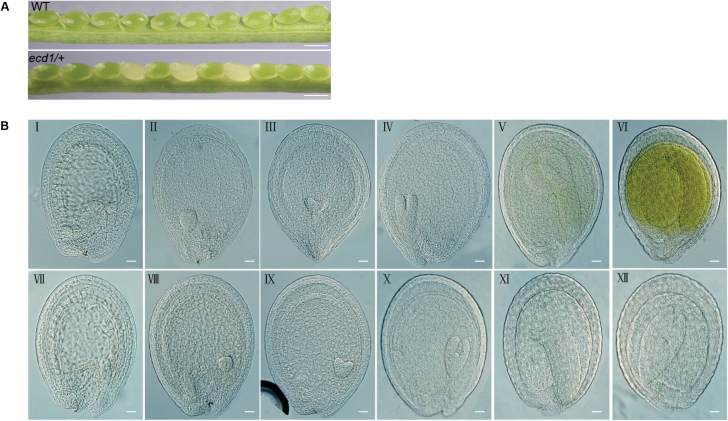
Embryogenesis of wild type and *ecd1/+* embryos. (A) A heterozygous *ecd1/*+ mutant silique showing that approximately one-quarter of the ovules are albino compared with the wild-type (WT). Scare bars are 0.5 mm. (B) I–VI, normal embryos of the wild-type: I, pre-globular; II, globular; III, heart-shaped; IV, torpedo-shaped; V, cotyledon; VI, mature. VII–XII, embryos of *ecd1*/+. VII–IX, embryos are similar to the wild-type; X–XII, development is arrested, with v-shaped embryos with wide, stunted cotyledons and no hypocotyl. Scare bars are 20 μm.

To study the detailed mechanisms of chloroplast development, we obtained a series of T-DNA insertion lines from the Arabidopsis Biological Resource Center, the products of which are predicted to be candidates for chloroplast biogenesis factors. An embryo-lethal line CS16045 of the gene *AT3G49170*, designated as *ecd1* (originally called *emb2261*), attracted our attention for further investigation. Failure to identify any progeny homozygous for the *ecd1* mutant allele suggested that the mutation causes embryonic lethality. We dissected the developing siliques and assessed the seeds under a dissecting microscope. In wild-type siliques, all the ovules developed normally, while in the heterozygous *ecd1/+* siliques, some ovules were white (Fig. 1A). In 33 siliques from the heterozygous *ecd1/+* plants, 177 out of 726 ovules were white, making the ratio of white to green ovules 1:3 (χ^2^=0.166, *P*>0.05) (data not shown). In older siliques, the white ovules became shrunken and aborted.

To determine precisely the stage of embryogenesis during which the *ECD1* mutant arrested development, developing seeds at various stages from self-pollinated heterozygous plants were cleared and observed using differential interference contrast microscopy. The normal wild-type embryos underwent typical developmental stages, ranging from pre-globular, globular, heart-shaped, torpedo-shaped, through to cotyledon and maturity. However, in the heterozygous *ecd1/+* siliques, although the homozygous mutant embryos consistently initiated cotyledons and showed continued growth and cell division, beyond the heart stage the development of the embryos was significantly slower than that of the wild-type (Fig. 1B). Mutant embryos consistently failed to elongate, developing instead as v-shaped embryos with wide, stunted cotyledons and no hypocotyl (see also Cushing *et al.*, 2005).

### Knock-down of *ECD1* results in a cotyledon-specific albino phenotype

**Fig. 2.**
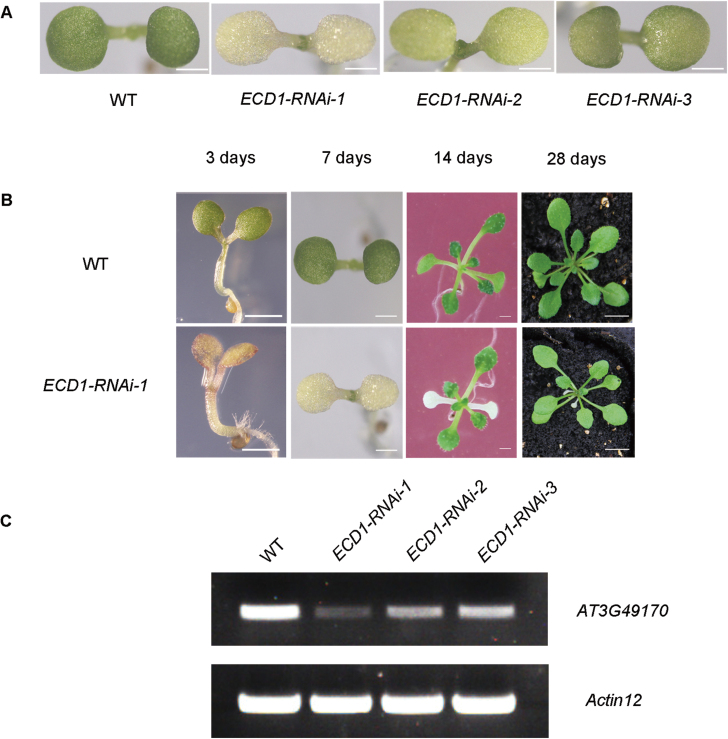
Characterization of the *ECD1*-RNAi transgenic plants. (A) Identification and isolation of RNAi lines with different degrees of inhibition of *ECD1* expression (WT: wild-type, ecotype Columbia). Plants were grown on MS medium with 2% (w/v) sucrose for 7 d. Scare bars are 1 mm. (B) Albino cotyledon phenotype of *ECD1*-RNAi-1 compared with the WT. Plants were grown on MS medium with 2% (w/v) sucrose (3–14 d) or soil (28 d). Scare bars are 1 mm for 3–14 d; 1 cm for 28 d. (C) Reverse transcription PCR (RT-PCR) using specific primers for *AT3G49170* or *Actin12* for 27 cycles for WT and RNAi lines with different degrees of inhibition of *ECD1*.

To further investigate the function of *ECD1*, we constructed RNAi lines. A total of 42 out of 76 RNAi-*ECD1* transgenic lines with the abnormal cotyledon phenotype were obtained (data not shown). In further studies, three RNAi transgenic lines with a range of stable phenotypes with respect to white cotyledons and stunted plant growth were selected (Fig. 2A, B); however, these lines all had true leaves that were normal green. RT-PCR showed that the phenotypes of these RNAi lines correlated with the expression levels of the *ECD1* gene (Fig. 2C). These results indicated that disruption of the *ECD1* gene led to abnormal cotyledons. The most severely affected line, *ECD1*-RNAi-1, in which the cotyledons were albino, was selected for further analysis.

### The *ECD1* gene encodes a chloroplast PPR protein belonging to the PLS subfamily

The *ECD1* gene encodes a putative protein of 850 amino acids with a predicated molecular mass of 95.5 kDa. The N-terminal 50 amino acids are predicated by ChloroP1.1 (http://www.cbs.dtu.dk/services/ChloroP/; last accessed 27 April 2018) to constitute a chloroplast transit peptide. Sequence analysis revealed that the ECD1 protein contains 17 PPR or PPR-like (P, L, and S) motifs, together with one E motif, one E+ motif, and one DYW motif in the C-terminal part (see Supplementary Fig. S1A). It belongs to the PLS subgroup of the PPR protein family. Protein alignment showed that ECD1 shares significant identity at the amino acid level with proteins from *Brassica*, grape (*Vitis*), eggplant (*Solanum*), *Zea mays*, and rice (*Oryza*) (Supplementary Fig. S1B).

**Fig. 3.**
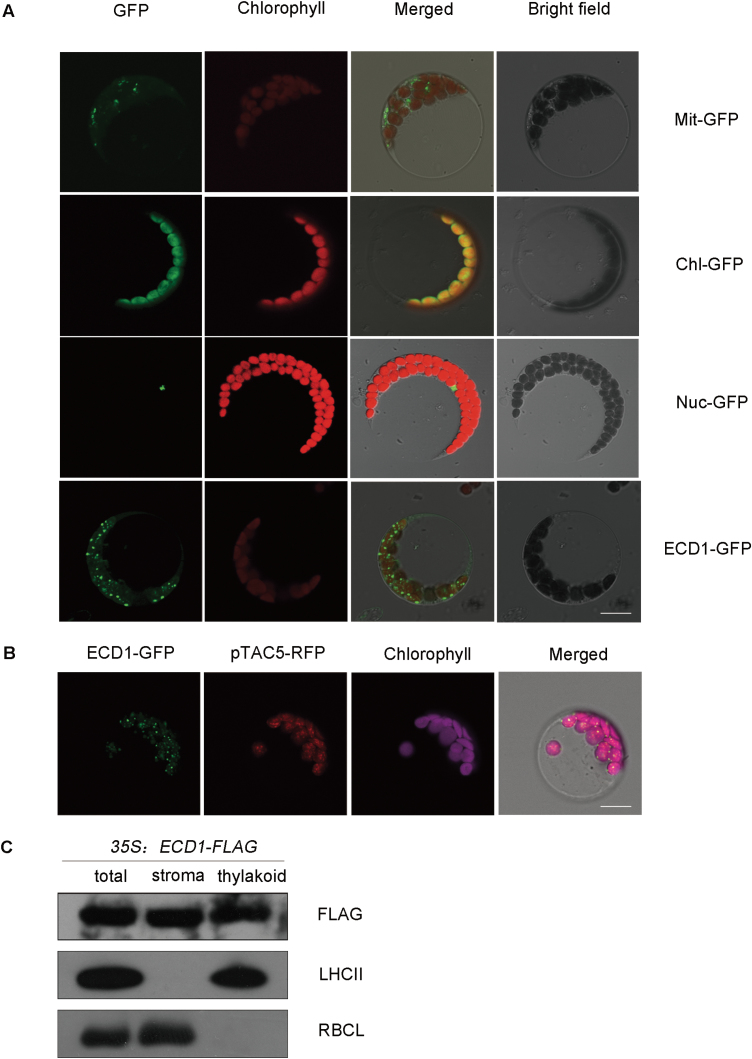
ECD1 is localized in the chloroplasts. (A) Subcellular localization of ECD1-GFP. The fluorescence of the ECD1-GFP fusion protein in protoplasts was observed using confocal laser scanning microscopy. Green fluorescence signals, chlorophyll red autofluorescence signals, and merged images are shown. The controls used for transformations are indicated to the right: Mit-GFP, control with mitochondrial localization signal of FROSTBITE1 (FRO1); Chl-GFP, control with the transit peptide of the Rubisco small subunit (RbcS); Nuc-GFP, control with nuclear localization signal of PTM-N (Sun *et al.*, 2011). The scare bar is 10 μm. (B) Co-localization of ECD1-GFP with the pTAC5-RFP protein in chloroplast nucleoids. The red fluorescence of chlorophyll has been adjusted for better contrast. The scare bar is 10 μm. (C) Immunoblot analysis of the ECD1-FLAG fusion protein in chloroplast subfractions. ECD1 localizes to both the stroma and thylakoid membrane fractions. Intact chloroplasts were isolated from *35S::ECD1-FLAG* transgenic seedlings and then separated into fractions. Polyclonal antisera were used to detect the ECD1-FLAG fusion protein, the light-harvesting complex II (LHCII), and the Rubisco large subunit (rbcL).

To determine the subcellular localization of the ECD1 protein, the 218 N-terminal amino acids were fused to the N terminus of synthetic GFP (sGFP). The ECD1-GFP fusion protein was transiently expressed in Arabidopsis protoplasts under the control of the cauliflower mosaic virus 35S promoter. We observed that the GFP fluorescence merged with the chlorophyll autofluorescence (Fig. 3A), indicating that ECD1 is a chloroplast protein. When ECD1-GFP was transiently co-expressed with red fluorescent protein (RFP) fused with pTAC5 (a well-characterized protein known to localize in nucleoids; Chi *et al.*, 2014), the green and red fluorescence signals within the chloroplasts were found to merge, indicating that ECD1 and pTAC5 were co-localized in chloroplast nucleoids (Fig. 3B). To further determine the localization of ECD1, intact chloroplasts of overexpressing ECD1-Flag transgenic plants were isolated and fractionated, and the proteins were separated by SDS-PAGE followed by immunoblot analysis using an anti-FLAG antibody. The ECD1 protein was detected in both the stromal and thylakoid fractions (Fig. 3C).

### Gene expression pattern of *ECD1*

To investigate the expression pattern of the *ECD1* gene in Arabidopsis, we made transgenic plants expressing the GUS protein under the control of the *ECD1*. The highest expression levels were observed in the cotyledons of seedlings. In addition, GUS activity was also detected in rosette leaves, flower buds, flowers, and siliques, with minimal expression also observed in roots (Supplementary Fig. S2). In the flowers, GUS staining was observed exclusively in green tissues, such as sepals, stamens, and carpels, but not in petals. Taken together, these findings showed that *ECD1* was widely expressed throughout the plant, but the highest expression was in the cotyledons, which was consistent with the albino cotyledon phenotype of the *ECD1*-RNAi transgenic plants. The results also indicated that *ECD1* expression is developmentally controlled and corresponds to early chloroplast development in seedlings.

### The *ECD1* mutation affects early chloroplast development in seedlings

Together with the chloroplast localization of the ECD1 protein, the albino cotyledons of *ECD1*-RNAi-1 plants suggested that the function of *ECD1* is related to early chloroplast development in seedlings. We next examined chloroplast morphology and ultrastructure in the transgenic plants using TEM. The wild-type chloroplast ultrastructure was similar in cotyledons and true leaves, with well-developed thylakoid membranes composed of grana connected by stroma lamellae (Supplementary Fig. S3). The chloroplasts of albino cotyledons of *ECD1*-RNAi-1 had no organized thylakoid membrane system but instead contained a large number of round or oblong membrane-bound internal vesicles. However, the chloroplasts of true leaves from *ECD1*-RNAi-1 contained organized thylakoid membranes similar to those of the wild type. The results indicated that *ECD1* is involved in chloroplast development in seedlings.

### The *ECD1* mutation affects the accumulation of proteins of photosynthetic complexes

**Fig. 4.**
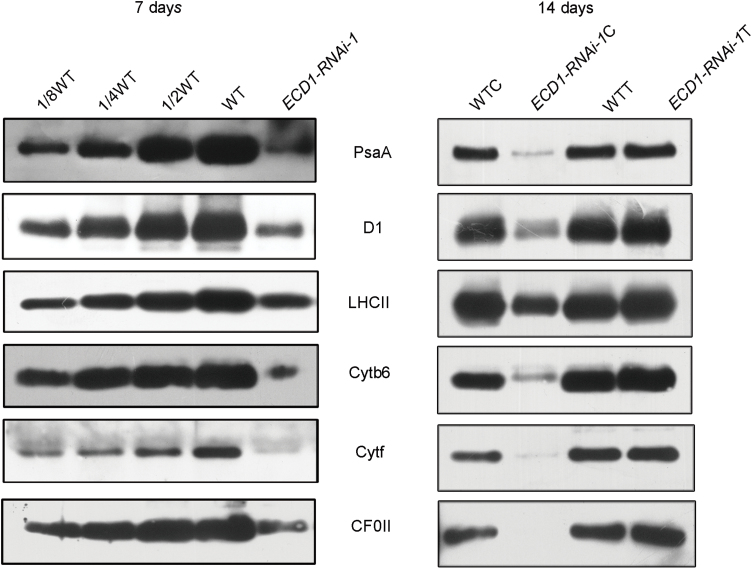
Immunoblot analysis of photosynthetic proteins. Total protein was separated by 10% Tricine/SDS-PAGE, electro-blotted, and probed using specific anti-PsaA, anti-D1, anti-LHCII, anti-Cytb6, anti-Cytf, and anti-CF0II antibodies. C and T refer to the proteins in cotyledons and true leaves, respectively, of *ECD1*-RNA-1 and wild-type (WT) seedlings.

Since the chloroplast ultrastructure was affected in the *ECD1*-RNAi transgenic plants, we investigated whether the accumulation of proteins of the photosynthetic complexes was different in these plants. Immunoblotting was performed to analyse the levels of the proteins of each of the thylakoid protein complexes. The protein levels of PSI (PsaA), PSII (D1 and LHCII), Cytb6/f (Cytb6 and Cytf), and ATPase (CF0II) of cotyledons in *ECD1-RNAi-1* were dramatically decreased compared to those of the wild type (Fig. 4). However, the accumulation of these photosynthetic proteins in true leaves of *ECD1-RNAi-1* was similar to that in the wild-type.

### *ECD1* is required for RNA-editing of *rps14*-149 in plastids of cotyledons

**Fig. 5.**
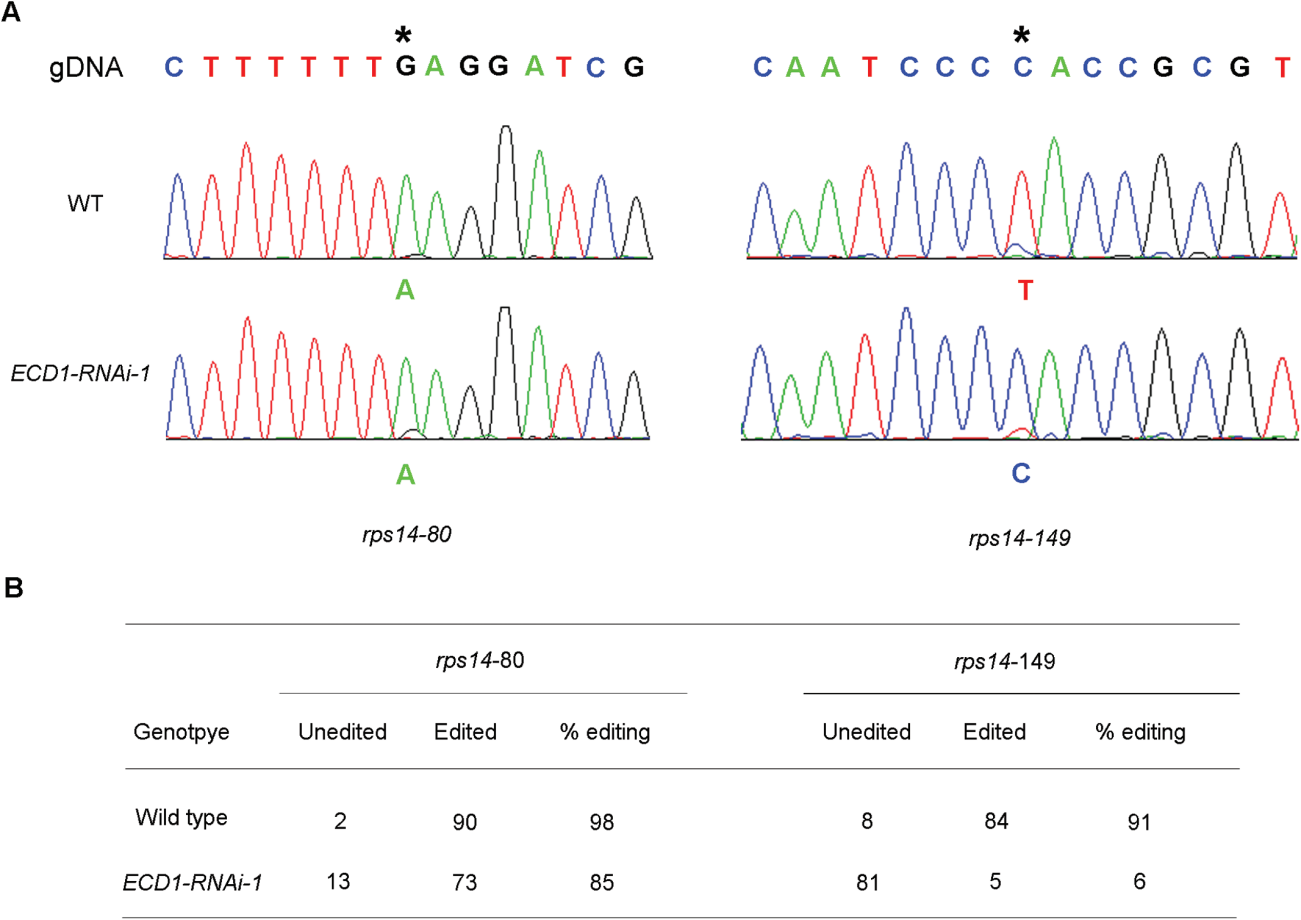
Analysis of RNA-editing of *rps14* transcripts from wild-type (WT) and *ECD1*-RNAi-1 seedlings. (A) RT-PCR products containing the *rps14*-80 and *rps14*-149 editing sites were directly sequenced. The editing sites of *rps14*-80 and *rps14*-149 are indicated by asterisks above the corresponding peaks. (B) The editing efficiency was determined by analysis of approximately 90 independent clones from RT-PCR products for *rps14*-80 and *rps14*-149 in the wild-type and *ECD1*-RNAi-1 line.

Previous studies have shown that the critical function of the DYW domain is involved in RNA editing. To test whether the RNA-editing status in the *ECD1*-RNAi transgenic plants was altered, we sequenced the 34 known editing sites in plastids of cotyledons and true leaves in the wild-type and transgenic plants; however, we did not include the nine sites recently found by Ruwe *et al.* (2013) because of their extremely low editing efficiency (<10%) even in the wild-type. The results showed that the editing efficiency of the ribosomal protein *rps14*-149 decreased to extreme low levels in the cotyledons of *ECD1*-RNAi-1 compared to that of the wild-type (Fig. 5). Multiple other sites were also affected to varying degrees (Supplementary Fig. S4), but no significant differences in the editing efficiency were detected in true leaves between *ECD1*-RNAi-1 and the wild-type (Supplementary Fig. S6). To test whether the editing deficiency was indirectly caused by the albino cotyledon phenotype, we pharmacologically induced albinism by using the plastid translation inhibitor lincomycin. Lincomycin treatment results in a severe albino phenotype and it has been reported to have severe effects on RNA editing (Tseng *et al.*, 2013). Our results showed that in lincomycin-treated seedlings, the editing efficiency of many sites was significantly reduced or even completely abolished, including *accD*-794, *accD*-58642, *petL*-5, *ndhB*-836, *ndhD*-878, and *ndhF*-290 and these were also affected in *ECD1*-RNAi-1 (Supplementary Fig. S4). However, no obvious editing deficiency of *rps14*-149 was detected in lincomycin-treated seedlings (Supplementary Figs S4, S5). To rule out the possibility that the editing defects were unique to lincomycin, we evaluated the effect of another inhibitor of chloroplast translation, spectinomycin, on editing and obtained similar results (Figs S4, S5). We therefore concluded that the mutation of *ECD1* specifically affected RNA editing of *rps14*-149 in plastids of cotyledons.

**Fig. 6.**
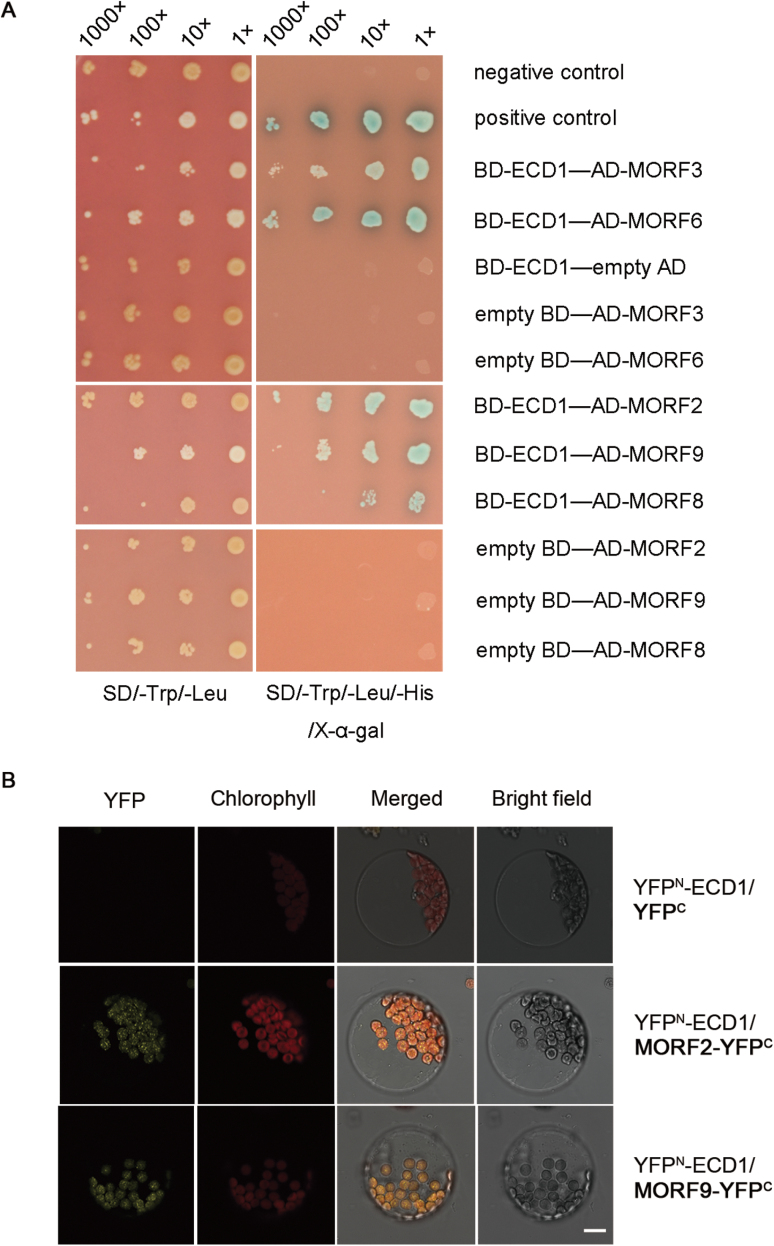
ECD1 interacts with multiple organellar RNA-editing factor (MORF) proteins. (A) Yeast two-hybrid screens with ECD1 showing that it interacts with MORF proteins. ECD1 was used as bait (BD), MORFs were used as prey (AD). The left panel shows the growth test on permissive medium lacking Trp and Leu; the right panel shows the same clones on selective medium lacking Trp, Leu, and His, and containing 40 μg ml^–1^ X-α-Gal (5-bromo-4-chloro-3-indolyl-a-D-galactopyranoside). Yeast cells transformed with pGBKT7-53 and pGADT7-T were used as positive controls and cells transformed with pGBKT7-lam and pGADT7-T were negative controls. (B) Bimolecular fluorescence complementation (BiFC) assays showing that YFP^N^-ECD1 interacts with MORF2-YFP^C^ (or MORF9-YFP^C^) to produce YFP fluorescence in the chloroplasts. The scale bar is 10 μm.

Yeast two-hybrid screening with ECD1 as bait identified several members of the family of multiple organellar RNA-editing factors (MORF) including MORF2, MORF3, MORF6, MORF8, and MORF9 (Fig. 6A). We also confirmed the interactions between ECD1 and the chloroplast-targeted proteins MORF2 and MORF9 through BiFC assays (Fig. 6B). The interaction with MORF proteins confirmed the effect of *ECD1* on RNA editing.

### ECD1 specifically interacts with the *cis*-elements of *rps14*-149 *in vitro* and *in vivo*

**Fig. 7.**
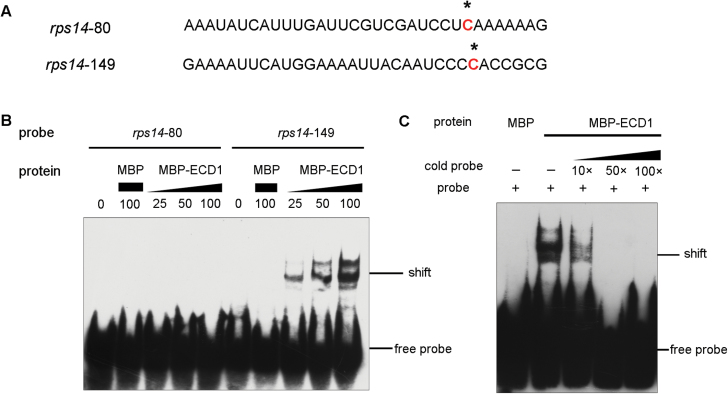
Gel electrophoretic mobility shift assays (EMSAs) with the *rps14* oligoribonucleotides. (A) The sequences used as oligonucleotide probes. Asterisks indicate the editing sites. (B) EMSA showing that MBP-ECD1 binds to the sequence around *rps14*-149, but not *rps14*-80. Increasing concentrations of protein (25, 50 and 100 nM) were incubated with 10 nM probes. The positions of the shift and free probes are indicated. (C) Unlabeled oligoduplexes with 10-fold, 50-fold, and 100-fold excess were used for competition to confirm the specific interaction between ECD1 and the *cis*-element around *rps14*-149.

There are two editing sites in *rps14* transcripts, *rps14*-80 and *rps14*-149. In cotyledons of the *ECD1*-RNAi transgenic plants, editing of *rps14*-149 was decreased dramatically, while that of *rps14*-80 remained normal. If the mutation of *ECD1* specifically affects RNA-editing of *rps14*-149, then ECD1 should bind to a *cis*-element surrounding this editing site. To analyse the ability of ECD1 to interact with such a *cis*-element, electrophoretic mobility shift assays (EMSAs) were performed. The recombinant ECD1 protein with an N-terminal MBP tag was expressed (Supplementary Fig. S7) and two oligoribonucleotides of 33 residues surrounding the editing sites of the *rps14*-80 and *rps14*-149 transcripts were synthesized for analysis by EMSA (Fig. 7A). As shown in Fig. 7B, binding of MBP-ECD1 to the *rps14*-149 oligonucleotide was increased with increasing concentrations of the MBP-ECD1 protein. The specificity of binding was confirmed using the same unlabeled oligoribonucleotide as a competitor (Fig. 7C), In contrast, no binding between MBP-ECD1 and the *rps14*-80 oligonucleotide was observed (Fig. 7B).

**Fig. 8.**
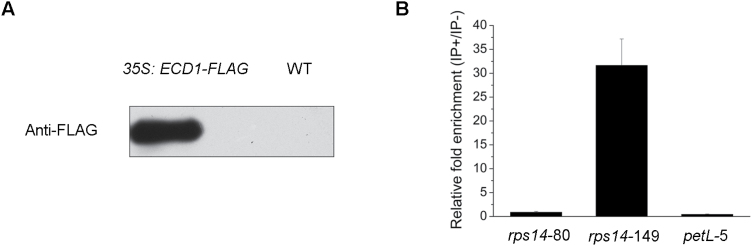
RNA immunoprecipitation analysis of ECD1 and *rps14*-149. (A) ECD1-FLAG protein accumulation in the *35S::ECD1-FLAG* transgenic seedlings compared with the wild-type (WT). (B) RNA immunoprecipitation analysis. IP+, anti-FLAG immunoprecipitation; IP-, mock immunoprecipitation. *rps14*-80, *rps14*-149, and *petL* are fragments that contain the editing sites of *rps14*-80, *rps14*-149, and *petL*, respectively. Data are means (±SE) obtained from three replicates.

To further test the ECD1–RNA interaction *in vivo*, RNAs co-immunoprecipitated with the anti-FLAG antibody were analysed by qRT-PCR using primers for transcripts containing the editing sites of *rps14*-149, and including transcripts containing *rps14*-80 and *petL* as controls. We detected enrichment fragments of *rps14*-149 in the anti-FLAG immupoprecipitate, but not of *rps14*-80 and *petL* (Fig. 8). This analysis suggested that ECD1 also binds to a *cis*-element surrounding *rps14*-149 *in vivo*.

### *ECD1* is indispensable for functional ribosomes in plastids

**Fig. 9.**
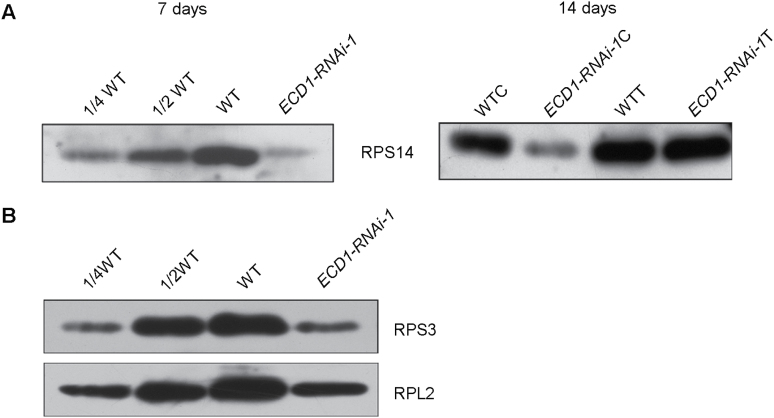
Immunoblot of chloroplast ribosomal subunits in the wild-type (WT) and *ECD1*-RNAi-1 line. (A) Immunoblots of PRS14 in 7- and 14-d-old seedlings. C and T refer to the proteins in cotyledons and true leaves, respectively, in the 14-d samples. (B) Immunoblot of RPS3 and RPL2 in samples of whole seedlings at 7 d old.

*rps14* encodes the ribosomal protein S14 and is essential for survival of tobacco plants (Tiller and Bock, 2014). Given the extremely low editing efficiency of *rps14*-149 and the decreased accumulation of photosynthesis proteins in the *ECD1*-deficient mutant, we examined the levels of the RPS14 protein. The level in the *ECD1*-RNAi transgenic plants decreased to less than one-quarter of that in the wild-type in the cotyledons, but there was no change in the true leaves (Fig. 9A). RPS14 is required for the accumulation of ribosomal 30S subunits. To determine whether the reduced content of RPS14 had any effects on ribosome levels, we examined the protein amounts for two other plastid-encoded ribosomal proteins, RPS3 and RPL2. by immunoblot analysis. Both proteins were decreased significantly in the *ECD1-RNAi-1* line (Fig. 9B). Thus, the defect in the accumulation of RPS14 protein in the *ECD1*-RNAi transgenic plants may have compromised ribosome accumulation, and the deficiency in translation was presumably responsible for the albino phenotype of cotyledons.

### The *ECD1* mutation affects plastid gene expression and plastid rRNA processing

**Fig. 10.**
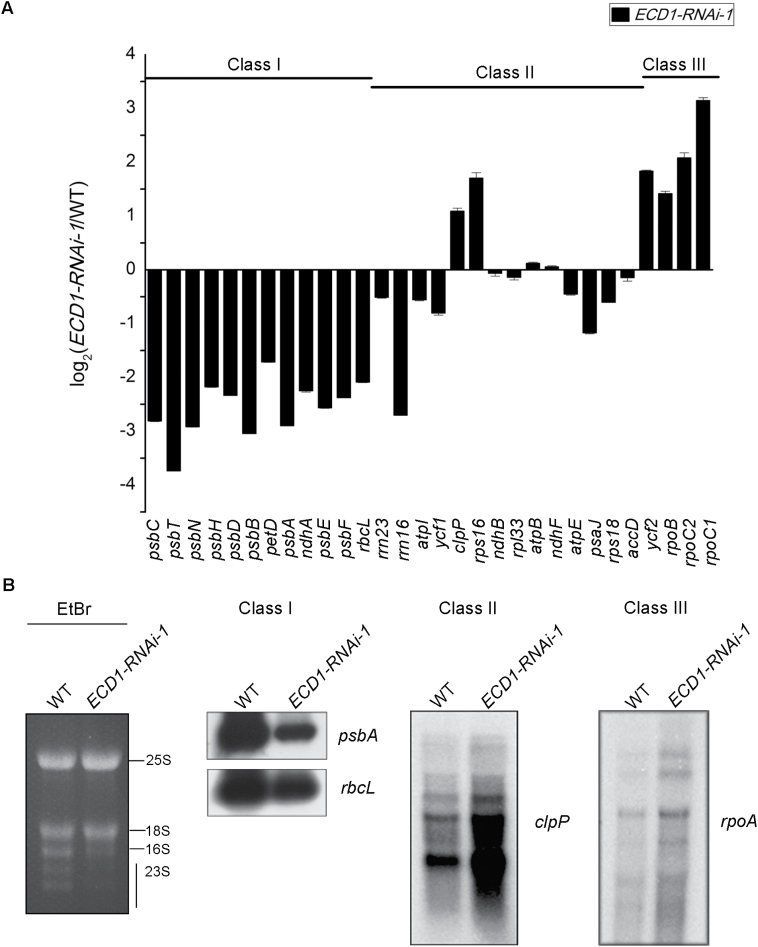
Chloroplast gene expression in *ECD1*-RNAi-1 relative to the wild-type (WT). (A) Transcript levels of chloroplast genes (Classes I–III) were measured by quantitative RT-PCR (qRT-PCR). Data are given as log_2_ of *ECD1*-RNAi-1/WT ratios from at least three independent experiments. Class I genes refer to *psbC*, *psbT*, *psbN*, *psbH*, *psbD*, *psbB*, *petD*, *psbA*, *ndhA*, *psbE*, *psbF*, and *rbcL*. Class II genes refer to *rrn23*, *rrn16*, *atpI*, *ycf1*, *clpP*, *rps16*, *ndhB*, *ndhF*, *atpE*, *psaJ*, and *rps18*. Class III genes refer to *accD*, *ycf2*, *rpoB*, *rpoC2*, and *rpoC1*. (B) RNA blot analysis of transcript levels for the different chloroplast gene classes. An ethidium bromide-staining gel is shown as a loading control (EtBR).

The expression of chloroplast genes significantly impacts on chloroplast development. We examined the transcript abundance of various chloroplast genes in 7-d-old seedlings by qRT-PCR. The results showed that the transcript levels of Class I genes (transcribed preferentially by PEP) were significantly reduced in *ECD1*-RNAi-1 compared with the wild-type (Fig. 10A). In contrast, transcript levels of Class III genes (transcribed preferentially by NEP) were either increased or unchanged, while Class II genes (transcribed by both NEP and PEP) were differentially regulated in *ECD1*-RNAi-1. The transcript abundance of other chloroplast genes that are not clearly classified are shown in Supplementary Fig. S8. In order to verify these results, we carried out RNA blot analysis of the *psbA*, *rbcL*, *clpP*, and *rpoA* genes using sequence-specific labeled probes. The steady-state levels of transcripts were in almost complete agreement with the qRT-PCR analysis, and the processing patterns between the wild-type and *ECD1*-RNAi-1 plants did not differ greatly (Fig. 10B, Supplementary Fig. S9). These results indicated that *ECD1* is essential for PEP but not for NEP activity.

**Fig. 11.**
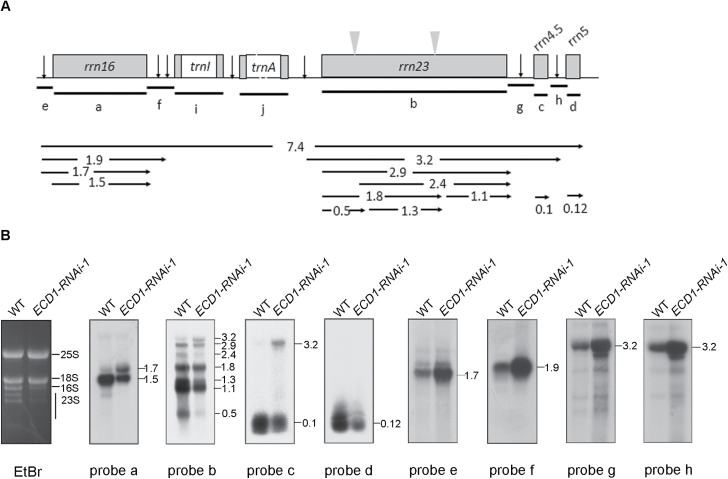
Expression and processing of chloroplast rRNA. (A) Schematic representation of the chloroplast *rrn* operon of Arabidopsis. The locations of probes (a–j) used for the RNA gel blot analysis and the size of transcripts (in kb) are indicated below the operon. (B) RNA gel blot analysis. Mature transcripts were decreased and the precursors were increased in *ECD1*-RNAi-1 compared to the wild-type (WT). RNA was extracted from 7-d-old seedlings. An ethidium bromide-staining gel is shown as a loading control (EtBr).

Decreased levels of rRNAs were found in the cotyledons of the *ECD1*-RNAi transgenic plants using ethidium bromide-stained agarose gel assays (Fig. 10B, Supplementary Fig. S9). We next examined the role of *ECD1* in rRNA metabolism. Chloroplast ribosomal RNAs are co-transcribed as a single RNA precursor that contains 16S, 23S, 4.5S, and 5S rRNAs, as well as two tRNAs (Fig. 11A). The precursor transcript undergoes a complex series of processing events before maturation. The 23S–4.5S bi-cistronic RNA (3.2 kb) undergoes endonucleolytic cleavage to produce a mature 4.5S rRNA and a 23S precursor (2.9 kb), which undergoes further maturation and ultimately generates three species of 1.1, 1.3, and 0.5 kb. The 16S precursor RNA (1.7 kb) is processed to a 1.5-kb mature 16S rRNA. RNA gel blot analysis was performed on the total leaf RNAs from 7- and 14-d-old wild-type and *ECD1*-RNAi-1 plants using rRNA-specific probes (indicated in Fig. 11). Higher transcript levels of the 3.2-kb 23S–4.5S rRNA precursor and the 1.7-kb 16S rRNA precursor were detected in the cotyledons of *ECD1-RNAi-1*, whereas the levels of the 1.5-kb 16S, 0.5-kb 23S, 0.1-kb 4.5S, and 0.12-kb 5S mature rRNAs decreased drastically (Fig. 11B). However, no obvious differences in the levels of the rRNA transcripts between the wild-type and *ECD1*-RNAi-1 were observed in true leaves (Supplementary Fig. S10). On the other hand, the transcript levels of the two tRNAs encoded by this operon, *trnI* and *trnA*, accumulated to the same extent in *ECD1*-RNAi-1 and the wild-type cotyledons (Supplementary Fig. S11). These results suggested that *ECD1* may play an important role in plastid rRNA maturation.

## Discussion

Chloroplasts are organelles that perform photosynthesis. The development of a functional chloroplast is regulated by a large number of genetic factors, especially nuclear-encoded factors. Given their importance, extensive studies have been carried out focusing on the identification of nuclear genes essential for chloroplast development. However, numerous nuclear mutants that impact on chloroplast development can easily be overlooked because of their severe, often lethal phenotype, especially embryo-lethal mutants. Chloroplast development is closely related to embryo development and, as a consequence, eliminating biosynthetic functions within the chloroplast and interfering with expression of the chloroplast genome often results in embryo lethality in Arabidopsis (Bryant *et al.*, 2011). A set of 119 nuclear genes encoding chloroplast-localized proteins has been identified, including many PPR proteins (Bryant *et al.*, 2011). Disruption of these genes results in an embryo-defective phenotype, highlighting the importance of chloroplasts in embryogenesis. Here, we identified a novel PPR protein, ECD1, which is required for chloroplast development in seedlings. A complete deletion of *ECD1* resulted in embryo lethality, indicating that *ECD1* is indispensable for plant growth and development. Evidence suggests that *accD*, which encodes one subunit of a multimeric acetyl-CoA carboxylase required for fatty acid biosynthesis, is amongst the most important chloroplast genes required for embryo development in Arabidopsis (Bryant *et al.*, 2011; Parker *et al.*, 2016). Editing of *accD*-794 and *accD*-58642 both decreased in the *ECD1*-RNAi transgenic plants (Supplementary Fig. S4). However, the editing deficiency of these sites may have been caused indirectly by the albino phenotype since they were also affected in seedlings treated with lincomycin or spectinomycin.

In the *ECD1*-RNAi transgenic plants, the chloroplast ultrastructure in cotyledons exhibited abnormal morphology and the thylakoid membrane structure was perturbed, suggesting that the albino phenotype of cotyledons in these plants was probably due to developmentally defective chloroplasts. Proper accumulation of plastid ribosomal proteins is a prerequisite for assembling functional ribosomes and is necessary for chloroplast development. A deficiency of nuclear-encoded chloroplast factors required for the synthesis of plastid ribosomal proteins can compromise assembly and accumulation of chloroplast ribosomes. PPR4 is required for the trans-splicing of the plastid *rps12* transcript and consequently affects the accumulation of plastid ribosomes (Schmitz-Linneweber *et al.*, 2006). The maize chloroplast protein PPR103 stabilizes the 5′-end of processed *rpl16* mRNAs and a loss of plastid ribosomes was also detected in *ppr103* mutants (Hammani *et al.*, 2016).

In cotyledons of the *ECD1*-RNAi transgenic plants, the accumulation of RPS14 decreased dramatically compared with the wild-type, which may have been due to the editing defects in *rps14*-149. We found that ECD1 is able to bind to the *cis*-element of *rps14*-149 but not to the other editing site *rps14*-80, indicating that ECD1 specifically affects RNA-editing of *rps14*-149. RNA-editing defects result in amino acid changes that may directly alter protein function, or act by destabilizing the protein or by affecting its ability to form complexes with other proteins (Hammani *et al.*, 2009). RNA-editing of *rps14*-149 changes Pro to Leu. Since Pro tends to disrupt α-helices and thus leads to instability of proteins, editing of this site could restore an α-helix and stabilize the RPS14 protein (Sugita *et al.*, 2006). RPS14 is essential for the assembly of the ribosomal 30S subunit and contributes to the peptide environment of the peptidyl transferase center in *E. coli* (Brochier *et al.*, 2000), and is also essential in tobacco plastids (Ahlert *et al.*, 2003; Tiller and Bock, 2014). In *Physcomitrella patens*, reduction of RNA editing in *rps14*-*C2* impairs the translation of the RPS14 protein and affects the function of the chloroplast ribosome, which then results in a pale-green phenotype and decreased photosynthetic activity in *PpPPR-45*-RNAi plants (Ichinose *et al.*, 2014). Thus, the decrease of RPS14 may block the proper assembly of plastid ribosomes in Arabidopsis. A deficiency in plastid ribosomes may account for the global defects in PEP-dependent transcripts, as translation of core subunits of the plastid-encoded RNA polymerase decreases to a level that is not sufficient for transcribing the PEP-dependent genes (Hess *et al.*, 1993; Zubko and Day, 2002; Chi *et al.*, 2008). As ribosome assembly and pre-rRNA processing are intimately linked, defects in rRNA processing in the cotyledons of *ECD1*-RNAi-1 may also be the consequence of the deficiency in ribosome assembly (Charollais *et al.*, 2003; Granneman and Baserga, 2005). In addition, the decreased accumulation of RPL2 and RPS3 provides further evidence for a deficiency of chloroplast ribosomes. Taken together, insufficient accumulation of plastid ribosomes may be the cause for the developmentally defective chloroplasts in cotyledons of *ECD1*-RNAi-1.

In the *ECD1*-RNAi transgenic plants, chloroplast development within cotyledons (but not in true leaves) was severely impaired, leading to the formation of white cotyledons. In recent years, several mutants with a phenotype of cotyledon-specific impairment have been isolated. *wco* mutants have a specific defect in 16S rRNA maturation in a cotyledon-specific manner (Yamamoto *et al.*, 2000). *sco1* mutants are defective in the chloroplast elongation factor G, which not only affects chloroplast mRNA translation during chloroplast formation in cotyledons, but also other developmental processes such as germination and flowering (Albrecht *et al.*, 2006). The proteins affected by *sco2/cyo1* are required for protein folding and both of them have DnaJ-like zinc finger domains (Shimada *et al.*, 2007; Albrecht *et al.*, 2008). The expression of *ECD1* is not limited to cotyledons only; however, the higher amount in cotyledons and its specific role in RNA-editing of *rps14*-149 in cotyledons but not in true leaves suggest that *ECD1* is more important in cotyledons than in leaves and other organs. Since *ECD1*-RNAi-1 is not lethal, the editing of *rps14*-149 is not completely abolished. It is expected that more severe editing defects occur in the *ecd1* homozygotes, leading to an embryo-lethal phenotype.

In conclusion, the results of our study indicate that the PPR protein ECD1 is a site-specific factor for the *rps14*-149 RNA-editing site, and it is required for early chloroplast development in seedlings. A decrease in *ECD1* expression leads to editing defects of *rps14*-149 in cotyledons, which result in decreased accumulation of the RPS14 protein; this in turn leads to lower levels of plastid ribosomes in cotyledons, and thus to defects in chloroplast development. Decreased expression of PEP-dependent genes and defective plastid rRNA processing were also observed in *ECD1*-RNAi transgenic plants, which can largely be accounted for by limiting amounts of plastid ribosomes.

## Supplementary data

Supplementary data are available at *JXB* online.

Fig. S1. Motifs and sequence analysis of ECD1.

Fig. S2. Expression patterns of *ECD1* within seedlings.

Fig. S3. Ultrastructure of chloroplasts in *ECD1*-RNAi-1 and wild-type seedlings.

Fig. S4. RNA-editing efficiency of various target sites in plastids of cotyledons in *ECD1*-RNAi-1 and wild-type seedlings, and in seedlings treated with lincomycin or spectinomycin.

Fig. S5. RNA editing efficiency of *rps14* transcripts in seedlings treated with lincomycin or spectinomycin.

Fig. S6. Plastid RNA editing in true leaves of *ECD1*-RNAi-1 and wild-type seedlings.

Fig. S7. Purification of MBP-ECD1.

Fig. S8. Transcript levels of chloroplast genes for which the transcribing RNA polymerase is unknown.

Fig. S9. RNA gel blot analysis of chloroplast RNAs in cotyledons and true leaves of 14-d-old wild-type and *ECD1*-RNAi-1 seedlings.

Fig. S10. RNA gel blot analysis of rRNAs in cotyledons and true leaves of 14-d-old wild-type and *ECD1*-RNAi-1 seedlings.

Fig. S11. RNA gel blot analysis of *trnI* and *trnA* in cotyledons and true leaves of wild-type and *ECD1*-RNAi-1 seedlings.

Table S1. List of primers used in this study.

Supplementary Figures S1-S11 and Table S1
