# Supplementary material for: ECD1 functions as an RNA-editing trans-factor of rps14-149 in plastids and is required for early chloroplast development in seedlings
Source: J Exp Bot. 2018 Apr 10;69(12):3037–51. doi: 10.1093/jxb/ery139 (PMC5972661; doi:10.1093/jxb/ery139)
Supplement: Supplementary Figures S1-S11 and Table S1 [file ery139_suppl_supplementary_figures_s1-s11_and_table_s1.pdf]

A.

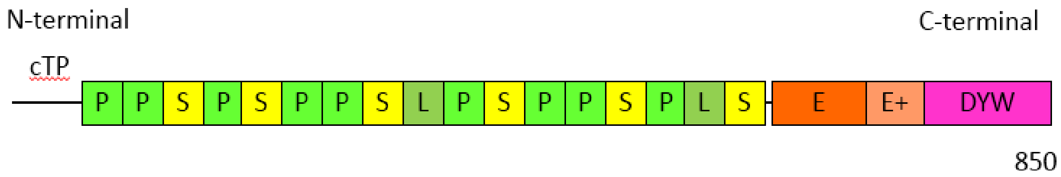

B.

```
Arabidopsis    1 MAMISFSFPSPAKLPIKSQPSVSN---RINVADRLILRHLNAGDLRGAVSALDLMARDGI
Brassica       1 MAMISFSLPSPAKLPVTSPPSVPN---RINIADRLILRHLNAGDLRGAISSLDLMARDGI
Vitis          1 --------------------SLSLKNPNFEPLKNRLIRQLDVGRLHHAFSTLDLMTQQNA
Solanum        1 --MITLSLPSPAKFIPPSSKSRRIRNPDFEALKDTLIRQANGGNLKQAISTLDQISQMGF
Zea            1 MATVRSTLPPSLPPPSPSSPPAHTLAATSRHRKPLT------------------TTAAGQ
Oryza          1 MALLPAKLPPPPATPSPSPPPPSSSTS-PRYPKRL-------------------AAHPAL

Arabidopsis   58 RPMDSVTFSSLLKSCIRARDFRLGKLVHARLIEF--DIEPDSVLYNSLISLYSKSGDSAK
Brassica      58 RPTDSATFSTLLKSCIRARDFRLGKLVHSRLAES--DIEPDSVLYNSLISLYSKSGDLAG
Vitis         41 PP-DLTTYSILLKSCIRFRNFQLGKLVHRKLMQS--GLELDSVVLNTLISLYSKCGDTET
Solanum       59 NP-DLTSYTVLLKSCIRTRNFQIGQLLHSKLNDS--PIQPDTIVLNSLISLYSKMGSWET
Zea           43 AALQPSEAAALLTAAARARDIHLGRALQGHLLRTGSLLETDAVVANSLLTLYSKCSAVAA
Oryza         41 SSNHPSEVSALLAAAARAGDLRLGRALHRRLLR-GDLLGRDAVVANSLLTLYSRCGAVAA

Arabidopsis  116 AEDVFETMRRFGKRDVVSWSAMMACYGNNGRELDAIKVFVEFLELGLVPNDYCYTAVIRA
Brassica     116 AEDVFETMGRIGKRDNVSWSAMMACYGNNGKELDAIKLFVGFLELGLVPNDYCYTAVIRA
Vitis         98 ARLIFEGMGN--KRDLVSWSAMVSCFANNSMEWQAIWTFLDMLELGFYPNEYCFAAVIRA
Solanum      116 AEKIFESMGE--KRDLVSWSAMISCYAHCGMELESVFTFYDMVEFGEYPNQFCFSAVIQA
Zea          103 ARSVFDGMP-VGLRDLVSWTAMASCLSRNGAEAEALRLFGETLEEGLLPNAFTLCAATQA
Oryza        100 ARNVFDGMR--GLRDIVSWTAMASCLARNGAERESLRLLGDMLESGLLPNAYTLCVAAHA

Arabidopsis  176 CSNSD-FVGVGRVTLGFLMKTGHFESDVCVGCSLIDMFVKGENSFENAYKVFDKMSELNV
Brassica     176 CSNPE-NVAVGRVILGFLMKTGYFESDVCVGCSLIDMFVKGENNLENAYKVFDQMSDLNV
Vitis        156 CSNAN-YAWVGEIIYGFVVKTGYLEADVCVGCELIDMFVKGSGDLGSAYKVFDKMPERNL
Solanum      174 CCSAE-LGWVGLAIFGFAIKTGYFESDVCVGCALIDLFAKGFSDLRSAKKVFDRMPERNL
Zea          162 CFASELFHLAGGAVLGLVFKLGFWGTDVSVGCALIDMFAKN-GDLVAMRRVFDGLFERTV
Oryza        158 CFPHELYGLTGGVILGLVHKMGFWGTDVSVGSALIDMLARN-GDLASARKVFDGLIEKTV

Arabidopsis  235 VTWTLMITRCMQMGFPREAIRFFLDMVLSGFESDKFTLSSVFSACAELENLSLGKQLHSW
Brassica     235 VTWTLMITRCMQMGFPKEAVRFFLDMVLSGFEADKFTLSSVFSACAELEDLFLGKQLHSW
Vitis        215 VTWTLMITRFAQLGCARDAIDLFLDMELSGYVPDRFTYSSVLSACTELGLLALGKQLHSR
Solanum      233 VTWTLMITRFSQLGASKDAVRLFLEMVSEGFVPDRFTFSGVLSACAEPGLSALGRQLHGG
Zea          221 VVWTLLITRYAQSGYSDEAVELFLDMLENGFQPDQYTLSSMLSACTELGSFRLGQQLHSL
```

```
Oryza        217 VVWTLLISRHVQGECAEEAVELFLNFLEEGFEPDRYTMSSMISACTELGSVSLGQQLHSL

Arabidopsis  295 AIRSGLVDD--VECSLVDMYAKCSADGSVDDCRKVFDRMEDHSVMSWTALITGYMKNCNL
Brassica     295 AIRSGMADD--VGCSLVDMYAKCSVDGSLDDCRKVFDRMEDHSVMSWTALITGYMQRCNL
Vitis        275 VIRLGLALDVCVGCSLVDMYAKCAADGSVDDSRKVFEQMPEHNVMSWTAIITAYVQSGEC
Solanum      293 VIKSRLSADVCVGCSLVDMYAKSTMDGSMDDSRKVFDRMADHNVMSWTAIITGYVQRGHY
Zea          281 ALRLGLESDSCVSCGLVDMYAKSHNGQSLHNAREVFNRMPKHNVMAWTALLSGYVQRGSQ
Oryza        277 VLRLGLASDGCVSCGLVDMYAKSHIKQSMEYANKVFERMPKHDVISWTALISGYVQCGVQ

Arabidopsis  353 ATEAINLFSEMITQGHVEPNHFTFSSAFKACGNLSDPRVGKQVLGQAFKRGLASNSSVAN
Brassica     353 DAEAINLFCEMISQGRVQPNHFTFSSAFKACGNLSDPRVGKQVLGHAFKRGLASNSSVAN
Vitis        335 DKEAIELFCKMIS-GHIRPNHFSFSSVLKACGNLSDPYTGEQVYSYAVKLGIASVNCVGN
Solanum      353 DMEAIKLYCRMID-GLVKPNHFTFSSLLKACGNLSNPAIGEQIYNHAVKLGLASVNCVAN
Zea          341 DNQVMILFCKMLN-EGIRPNHITYSSMLKACANLGDQDSGRQIHTHCVKSNLADLNVVGN
Oryza        337 ENKVMSLFGDMLN-ESIKPNHITYSSILKACAIISDQDSGRQVHAHVIKSNLDDVHIVGN

Arabidopsis  413 SVISMFVKSDRMEDAQRAFESLSEKNLVSYNTFLDGTCRNLNFEQAFKLLSEITERELGV
Brassica     413 SVISMFVKSDMMEDARRAFESLSEKNLVSYNTFLDGACRSLDFEEAFELFHEITERELGV
Vitis        394 SLISMYARSGRMEDARKAFDILFEKNLVSYNAIVDGYAKNLKSEEAFLLFNEIADTGIGI
Solanum      412 SLISMYAKSGRMEEARKAFELLFEKNLASYNIIVDGCSKSLDSAEAFELFSHI-DSEVGV
Zea          400 ALVSMYAESGSIEEARHAFDQLYEKNMVSFSGNLDGDGRSNTYQ-----DYQIERMELGI
Oryza        396 ALVSMYTESGSMEEARRVFTQLYEKSMSSLISE-----RRNAPV-----DHQIARMDMGI

Arabidopsis  473 SAFTFASLLSGVANVGSIRKGEQIHSQVVKLGLSCNQPVCNALISMYSKCGSIDTASRVF
Brassica     473 SAFTFASLLSGVASVGSIRKGEQLHSQVVKLGLSCNQPVCNALISMYSKCGSIDTASRVF
Vitis        454 SAFTFASLLSGAASIGAMGKGEQIHGRLLKGGYKSNQCICNALISMYSRCGNIEAAFQVF
Solanum      471 DAFTFASLLSGAASVGAVGKGEQIHSRVLKAGIQSSQSVCNALISMYSRCGNIEAAFQVF
Zea          455 STFTFGSLISAAASVGMLTKGQRLHALSLKAGFGSDRAIGNSLVSMYSRCGYLVDACQVF
Oryza        446 SSSIFASLISAAASVGMLTKGQQLHAMSLKAGFGSDRFVSNSLVSMYSRCGYLEDACRSF
```

```
Arabidopsis  533 NFMENRNVISWTSMITGFAKHGFAIRVLETFNQMIEEGVKPNEVTYVAILSACSHVGLVS
Brassica     533 NLMEDRNVISWTSMITGFAKHGFAKRVLETFNQMMEAGVKPNEVTYVAILSACSHVGLVS
Vitis        514 NEMEDRNVISWTSMITGFAKHGFATRALEMFHKMLETGTKPNEITYVAVLSACSHVGMIS
Solanum      531 EGMEDRNVISWTSIITGFAKHGFAHRAVELFNQMLEDGIKPNEVTYIAVLSACSHVGLVD
Zea          515 DEMNDHNVISWTSMISGLAKHGYAARALELFHDMIAAGVKPNDVTYIAVLSACSHAGLVK
Oryza        506 NELKDRNVISWTSMISGLAKHGYAERALTLFRAMMLAGVKPNDVTYIAVLSACSHVGLVM

Arabidopsis  593 EGWRHFNSMYEDHKIKPKMEHYACMVDLLCRAGLLTDAFEFINTMPFQADVLVWRTFLGA
Brassica     593 EGWRHFNSMYEDHKIKPKMEHYACMVDLLCRSGLLTDAFEFINTMPFQADVLVWRTFLGA
Vitis        574 EGQKHFNSMYKEHGIVPRMEHYACMVDLLGRSGLLVEAMEFINSMPLMADALVWRTLLGA
Solanum      591 EGWKYFDSMSIDHGITPRMEHYACMVDLLGRSGSLEKAVQFIKSLPLNVDALVWRTLLGA
Zea          575 EGKEHFRMMQKHHGLIPRMEHYACMVDLLGRSGLVEDALDFINEMPCQVDALVWKTLLGA
Oryza        566 EGKEYFRSMQRDHGLIPRMEHYACMVDLLARSGLVEEALEFISEMPLQADALVWKTLLGA

Arabidopsis  653 CRVHSNTELGKLAARKILELDPNEPAAYIQLSNIYACAGKWEESTEMRRKMKERNLVKEG
Brassica     653 CRVHSNTELGEIASRKILELDPNEPAAYIQLSNIYASTGKWEESAEMRKKMKERNLVKEG
Vitis        634 CRVHGNTELGRHAAEMILEQEPDDPAAYILLSNLHASAGQWKDVVKIRKSMKERNLIKEA
Solanum      651 CQVHGNLQLGKYASEMILEQEPNDPAAHVLLSNLYASRGQWEEVAKIRKDMKEKRMVKEA
Zea          635 CKTHNNMDIGEIAANHVIQLEPQDPAPYVLLSNLYAEAGLWDQVARIRSLMRDKNLMKEK
Oryza        626 CRTHDNIDIGEIAAKNVIELEPRDPAPYVLLSNLYADAGLWDEVARIRSAMRDKNLNKET

Arabidopsis  713 GCSWIEVGDKIHKFYVGDTAHPNAHQIYDELDRLITEIKRCGYVPDTDLVLHKLEEENDE
Brassica     713 GCSWIEVGDKFHKFYVGDTSHPNTHRIYDELDRLIREIKRCGYVPDTDLVLHKLEEEDDV
Vitis        694 GCSWIEVENRVHRFHVGETSHPQAWQIYQELDQLASKIKEMGYIPDTDFVLHDIEEE---
Solanum      711 GCSWMEAENSVHKFYVGDTKHPKAKEIYEKLNKVALKIKEIGYVPNTDLVLHEVEDE---
Zea          695 GLSWMHVDNTIHEFRAGDTSHPQAEEIYTKLETLIREIKVMGYVPDTSVVLHDMSDE---
Oryza        686 GLSWMEVENTTHEFRAGDTCHPRAQDIYAKLDTLVTEIKGMGYVPDTSIMLHDMSDD---

Arabidopsis  773 AEKERLLYQHSEKIAVAFGLIS-TSKSRPVRVFKNLRVCGDCHNAMKYISTVSGREIVLR
Brassica     773 AEKERLLFQHSEKIAVAFGLIS-TSKSRPVRVFKNLRVCGDCHTAMKYITVVSGREIVLR
Vitis        751 -QKEQFLFQHSEKIAVAFGLIS-TSQSKPIRIFKNLRVCGDCHTAIKYISMATGREIVVR
Solanum      768 -QKEQYLFQHSEKIALAFGLIS-TSKQKPIRIFKNLRVCGDCHNAMKFISVAEGREIIIR
Zea          752 -LKELCLLQHSEKIAVAFGLISCTSATKPIRIFKNLRVCVDCHSALKYVSKATGREIILR
Oryza        743 -LKEQYLLQHSEKIAVAFGLIA-TSAPKPIRIFKNLRVCADCHSAIKYMSKATGRVIILR

Arabidopsis  832 DLNRFHHFKDGKCSCNDYW
Brassica     832 DINRFHHFKDGKCSCNDYW
Vitis        809 DSNRFHHIKNGVCSCNDYW
Solanum      826 DSNRFHHIKDGLCSCNDYW
```

```
Zea      811 DSNRFHRMKDGECSCGEYW
Oryza    801 DSNRFHRMKDGECSCGEYW
```

Supplementary Figure S1. **Motifs and sequence analysis of ECD1.**

(A) Predicted motif structure of ECD1. Pentatricopeptide repeat (PPR) (or PPR-like), E, E+ and DYW motifs are depicted as boxes with letters. The designation of the P, L and S corresponds to the PPR motif, PPR-like S (for short) motif, and PPR-like L (for long) motif, respectively, proposed by Lurin et al. (2004). (B) Amino acid sequence alignment of ECD1 from various species. Strictly conserved amino acids are indicated in black, and closely relatedly residues are indicated in grey. The following sequences were used to build the alignment: *Brassica napus* (*Brassica*), CDY08114; *Vitis vinifera* (*Vitis*), CBI23556; *Solanum lycopersicum* (*Solanum*), XP_004231236; *Zea mays* (*Zea*), XP_008649476; *Oryza brachyantha* (*Oryza*), XP_006655097.

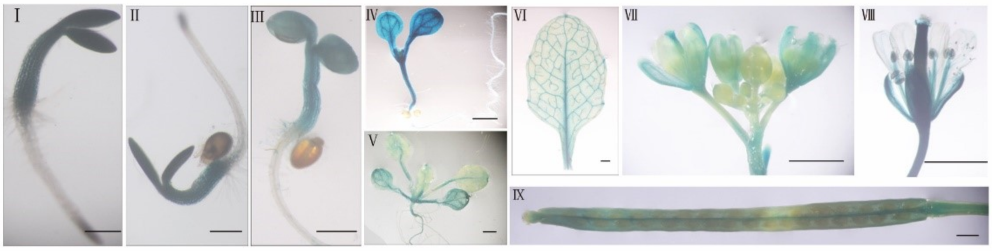

Supplementary Figure S2. Expression pattern of *ECD1*.

GUS expression was analyzed in *pECD1::GUS* transgenic plants. I, 1 DAG seedlings. II, 3 DAG seedlings. III, 5 DAG seedlings. IV, 7 DAG seedlings. V, 14 DAG seedlings. VI, rosette leaves. VII, Flower buds. VIII, Flowers. IX, Siliques. DAG: days after germination. Scare bars = 1mm.

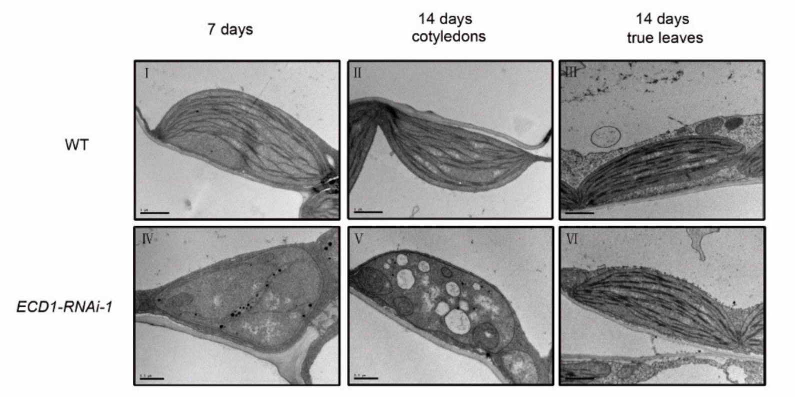

Supplementary Figure S3. **Ultrastructure of chloroplasts in *ECD1-RNAi-1* and wild-type.**

Wild-type chloroplast (I) and *ECD1-RNAi-1* chloroplast (IV) in cotyledons from 7-day-old plants. Wild-type (II) and *ECD1-RNAi-1* (V) chloroplast in cotyledons from 14-day-old plants. Wild-type (III) and *ECD1-RNAi-1* (VI) chloroplast in true leaves from 14-day-old plants. Scare bars = 1 μm in I, II, III and VI. Scare bars = 0.5μm in IV and V.

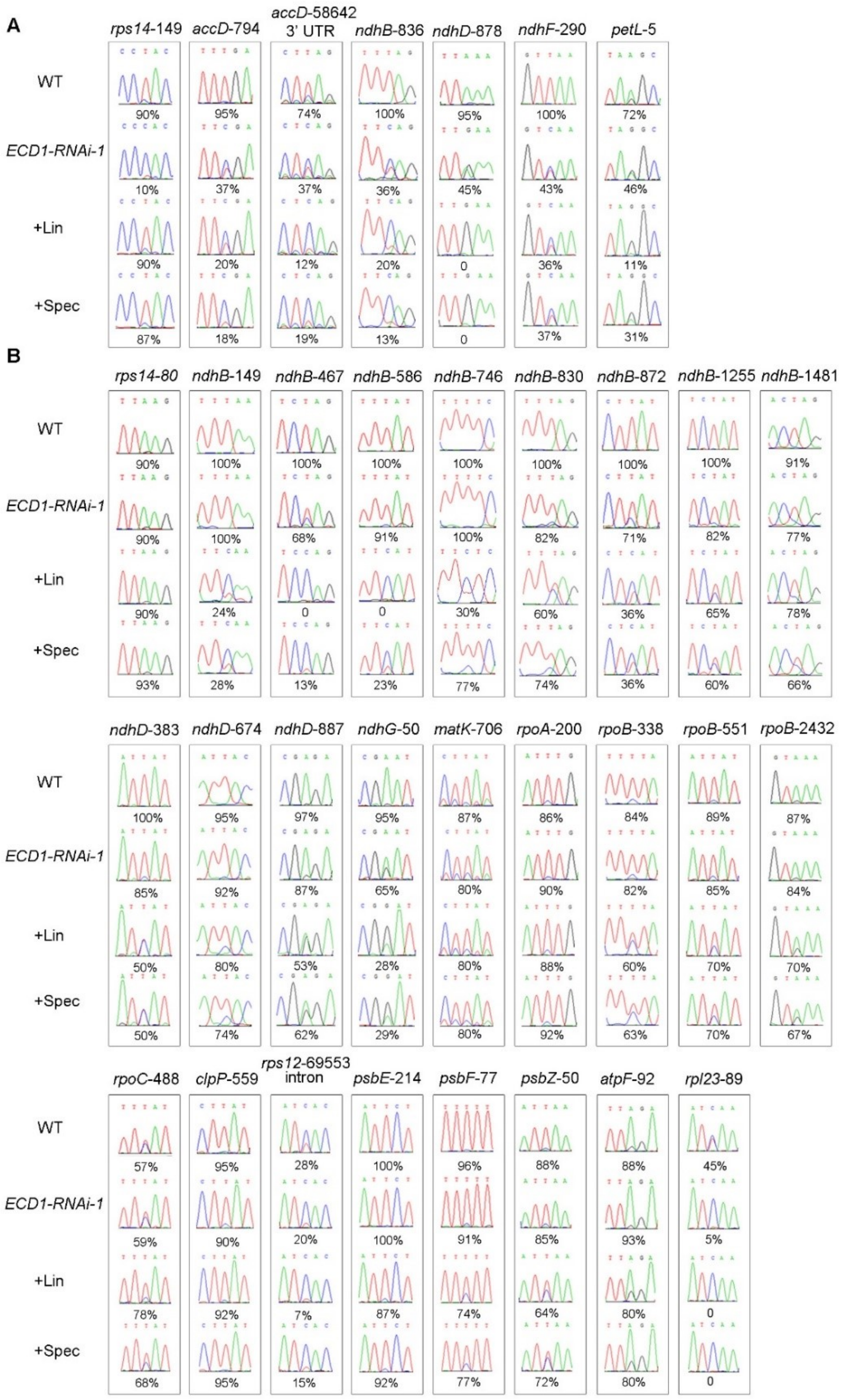

Supplementary Figure S4. RNA editing efficiency of various target sites in plastids of cotyledons.

Sequencing analysis showing the editing efficiency of the (A) affected sites and (B) unaffected or slightly affected sites in *ECD1-RNAi-1*. +Lin, the lincomycin treated seedlings. +Spec, the spectinomycin treated seedlings. RNA was extracted from 7-day-old seedlings.

A

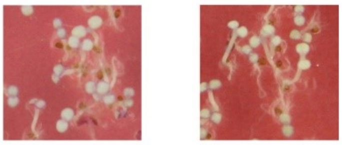

B

	rps14-80			*rps14*-149		
Genotype	Unedited	Edited	% editing	Unedited	Edited	% editing
+Lin	3	88	97	6	85	93
+Spec	3	71	96	6	68	92

Supplementary Figure S5. Analysis of RNA editing of *rps14* transcripts from the wild type seedlings treated by lincomycin (Lin) or spectinomycin (Spec).
(A) Phenotype of wild type seedlings grown on either 500µM Lin or 50mg/L Spec for 7d. (B) The editing efficiency was determined by analysis of more than 70 independent clones from RT-PCR products including *rps14*-80 and *rps14*-149 in the Lin or Spec treated seedlings.

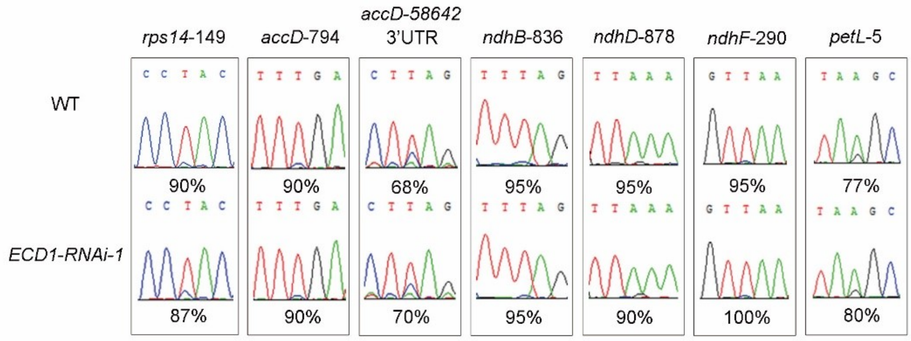

Supplementary Figure S6. Plastid RNA editing in true leaves of *ECD1-RNAi-1*. RNA editing efficiency of sites which are affected in cotyledons were normal in true leaves of *ECD1-RNAi-1*. RNA was extracted from true leaves of 14-day-old seedlings.

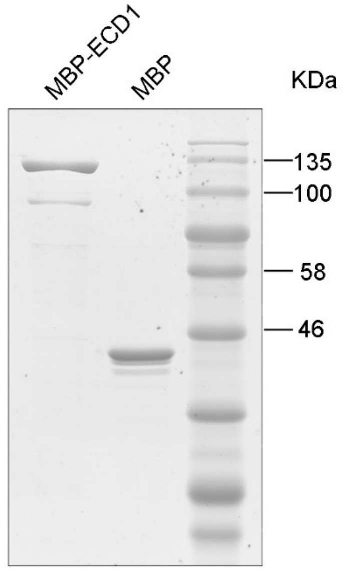

Supplementary Figure S7. Purification of MBP-ECD1.

Purified MBP-ECD1 and MBP proteins were analyzed by SDS-PAGE and the gel was stained with Coomassie Brilliant Blue. The predicted sizes of MBP-ECD1 and MBP are 134 and 44 KDa, respectively.

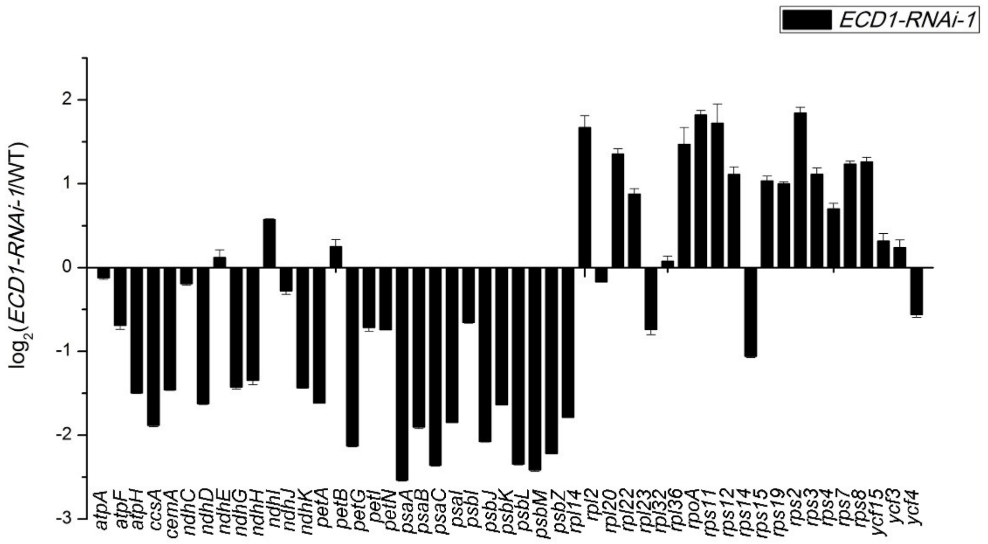

Supplementary Figure S8. Transcript levels of chloroplast genes for which the transcribing RNA polymerase is unknown.

Data are given as $\log_2$ of *ECD1-RNAi-1*/wild-type ratios from at least three independent experiments. RNA was extracted from 7-day-old seedlings.

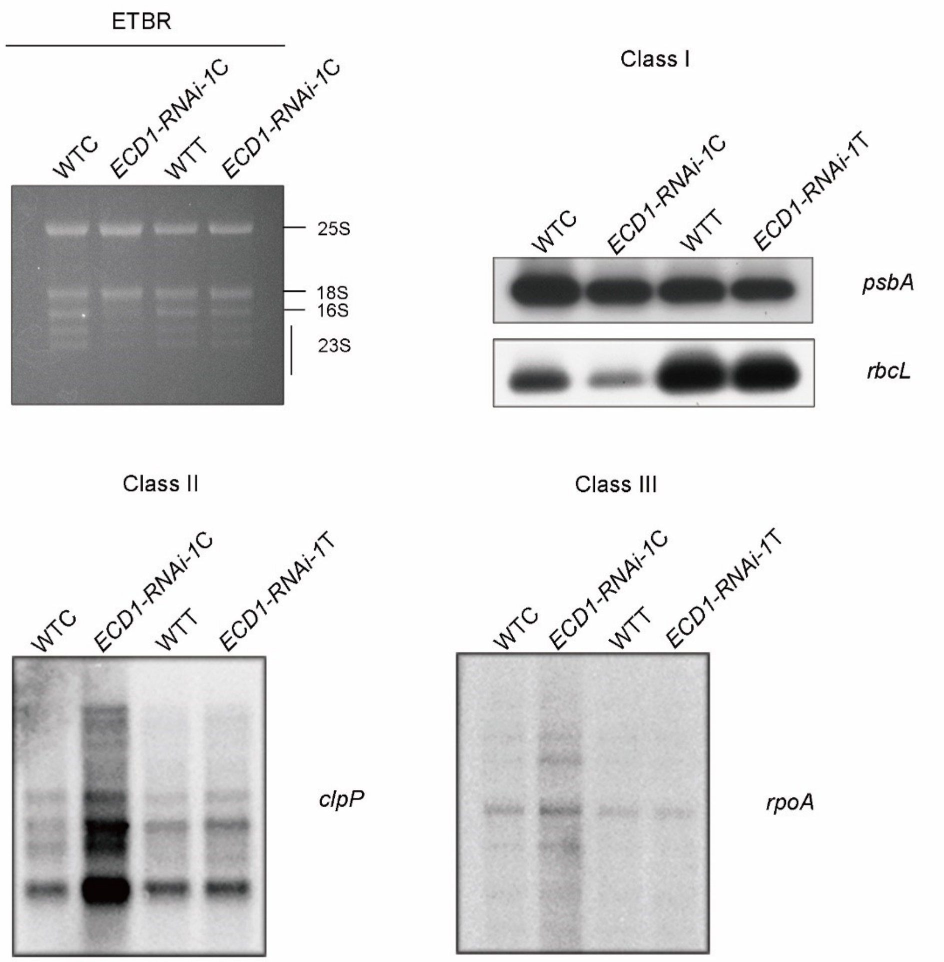

Supplementary Figure S9. **RNA gel blot analysis of chloroplast RNAs with 14-day-old WT and *ECD1-RNAi-1* seedlings.**

*ECD1-RNAi-1*C and *ECD1-RNAi-1*T refer to cotyledons and true leaves of *ECD1-RNAi-1*, respectively. An ethidium bromide-staining gel was shown as a loading control.

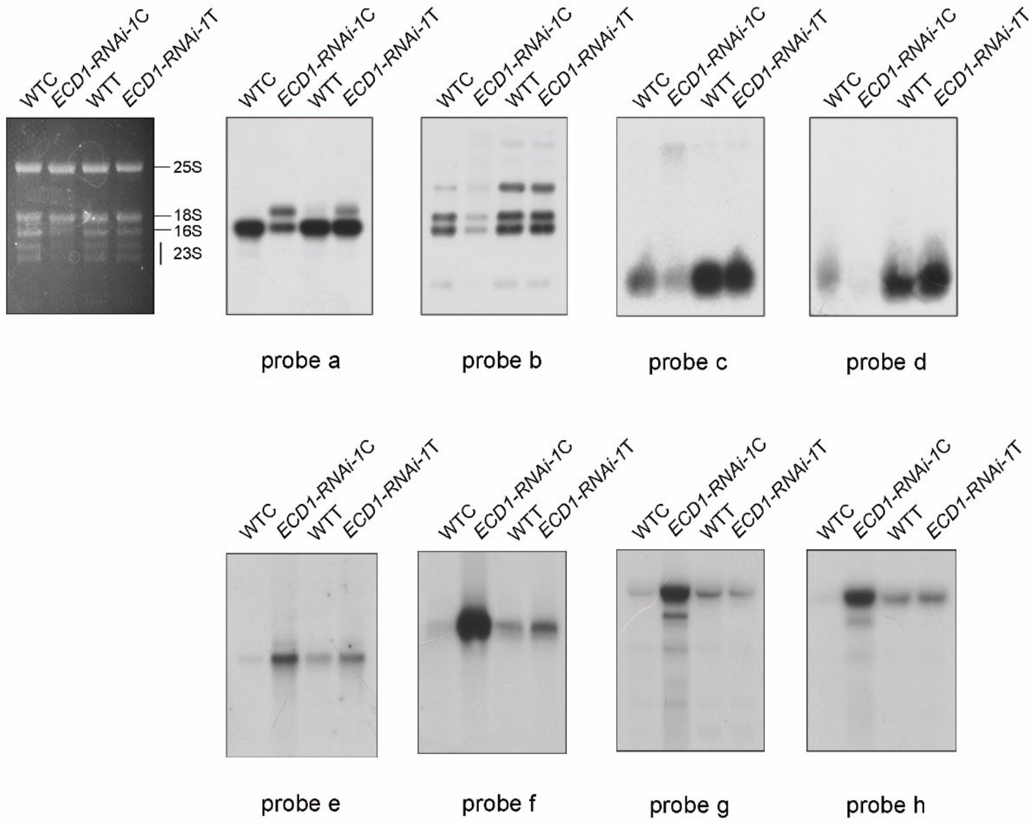

Supplementary Figure S10. RNA gel blot analysis of rRNAs with 14-day-old WT and *ECD1-RNAi-1* seedlings.

*ECD1-RNAi-1*C and *ECD1-RNAi-1*T refer to cotyledons and true leaves of *ECD1-RNAi-1*, respectively. Probes were indicated in figure 11A. An ethidium bromide-staining gel was shown as a loading control (left panel).

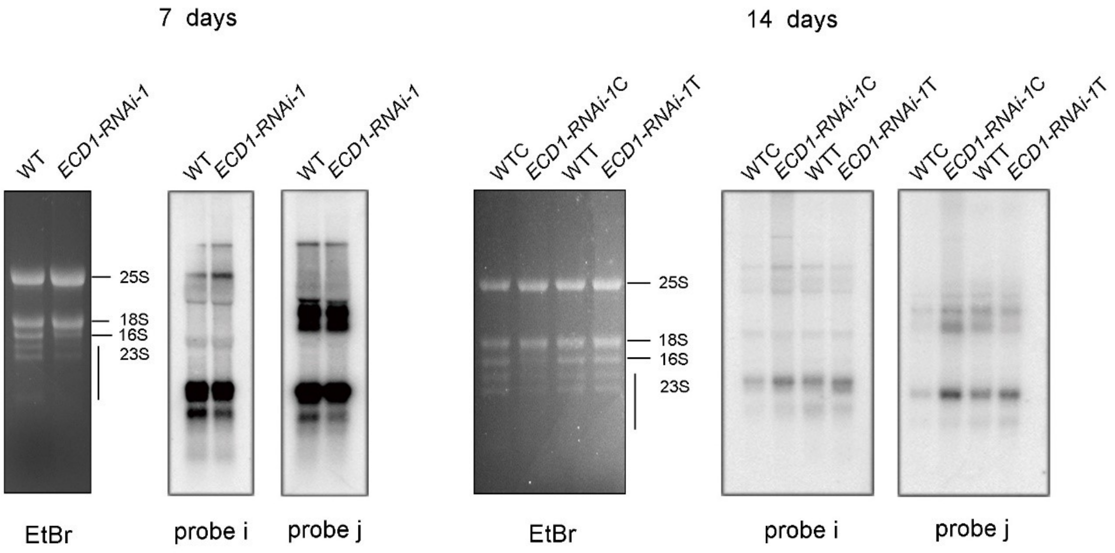

Supplementary Figure S11. RNA gel blot analysis of *trnI* and *trnA*.

*ECD1-RNAi-1*C and *ECD1-RNAi-1*T refer to cotyledons and true leaves of *ECD1-RNAi-1* respectively. Probes were indicated in figure 11A. An ethidium bromide-staining gel was shown as a loading control.

Table S1 List of primers used in this study.

Primer name	Sequences (5'-3')	Purpose
ndhB-1F	TTTGCTTCTCTTCGATGGAAG	RNA editing
ndhB-1R	ACGACTGGAGTGGGAGATCCTTC	RNA editing
ndhB-2F	CGTATACGAAGGATCTCCCAC	RNA editing
ndhB-2R	CCTGAGCAATCGCAATAATCG	RNA editing
ndhD-F	TTGAGTACGCGTTCTTTGGAC	RNA editing
ndhD-R	AATAGCTCCATTAAGTCCAGG	RNA editing
ndhF-F	AAAACCTTCGCCGCATGTGG	RNA editing
ndhF-R	GCATTCGCTGCAATAGGTCG	RNA editing
ndhG-F	ATGGATTTGCCTGGACCAATAC	RNA editing
nhdG-R	TTGATAAATGAATTCCTATTTGTTG	RNA editing
petL-F	AAATTTGGTAATTAACACGG	RNA editing
petL-R	ATTTCAATTGAAACTTAGGG	RNA editing
accD-1F	TTCATTTGTAGTGAAAGCGG	RNA editing
accD-1R	TTTCGCCTACTACGGATCCC	RNA editing
accD-2F	CTACTACCGGTGGAGTGACAGC	RNA editing
accD-2R	AGAATCTGATCTAACAACAGGGAA	RNA editing
rps14-F	TTATAGGGAGAAGAAGAGGC	RNA editing
rps14-R	TACCAGCTTGATCTTGTTGC	RNA editing
atpF-F	GAGTTTCGGATTTAATACCG	RNA editing
atpF-R	AGCTCCTTGTAAAGCTTGTTG	RNA editing
clpP-F	TTGGGTTGACATATACAACCG	RNA editing
clpP-R	TGAACCGCTACAAGATCAAC	RNA editing
matK-F	CGTTACCGGGTAAAAGATGC	RNA editing
matK-R	AGCGGCGTATCCTTTGTTGC	RNA editing
psbE-F	ACAGGAGAACGTTCTTTTGC	RNA editing
psbE-R	ATATAATCCATCCGAATGGG	RNA editing
psbF-F	ACAGGAGAACGTTCTTTTGC	RNA editing
psbF-R	ATATAATCCATCCGAATGGG	RNA editing
psbZ-F	ATGAGATACGCGATCCAGTATAC	RNA editing
psbZ-R	TCAAGAGATAAGAGAATTAAGGATAC	RNA editing
rpoA-F	GTAAGCGTCTTTATTATGGACGC	RNA editing
rpoA-R	CTTGATGAAGTGCTTCTTTAGGAG	RNA editing
rpoB-F	GAAAACCAGTAGGAATATGC	RNA editing
rpoB-R	GTCTCCAATTAATATTTCGGCG	RNA editing
rpoC1-F	AGTTTTGTGAACAATGTGGAGTTG	RNA editing
rpoC1-R	TGAATGATGGGTCTCAACTCGG	RNA editing
rps12-F	CTTGTACAATTCACATTCTTTGGC	RNA editing
rps12-R	ACAAGACAGCCAATCCGAAAC	RNA editing
rpl23-F	AAGAGGTGGAATAGAATAACCCG	RNA editing
rpl23-R	CAATTCCTACTGGATGCACGC	RNA editing

ECD1-RT-F	GCCCTTCCAAGCCGATGTTT	RT-PCR
ECD1-RT-R	CCGGTCTAGATTTCGACGTGCTTAT	RT-PCR
rps14-80-QF	GAAGAAGAGGCAAAAATTGGAA	RIP
rps14-80-QR	TTCTCACTTAGCGACGGAATC	RIP
rps14-149-QF	TCGCTAAGTGAGAAATGGAAAA	RIP
rps14-149-QR	CGTCGATGAAGACGTGTAGG	RIP
petL-QF	ATTTTATTGAGTCCCTTCATGC	RIP
petL-QR	AAAGCTGCTAGTAGAAAACCGAAA	RIP
ECD1-BiFC-F	CCCCGAGCTCATGGCGATGATAAGCTTTTCGTTTC	BiFC
ECD1-BiFC-R	CCCCCCGTCGACCCCAATAATCATTACAAGAACAT	BiFC
MORF2-BiFC-F	CTTTGAGCTCATGGCTTTGCCTTTGTCTGGCAC	BiFC
MORF2-BiFC-R	CTTTGTCGACCTCTTGTGTTTTCTCTGCGGC	BiFC
MORF9-BiFC-F	CCTTGAGCTCATGGCTTCCTTCACAACAACTTC	BiFC
MORF9-BiFC-R	CCTTGTCGACCAGAGGAATCAGAGGCTGCTG	BiFC
ECD1-Y2H-F	CCCCCATATGCTCATGGCTCGTGATGGGATTCGTC	Y2H
ECD1-Y2H-R	CCCCGAGCTCTCACCAATAATCATTACAAGAACAT	Y2H
MORF2-Y2H-F	CCTTGAATTCTGTGGAGCTAACCGGTC	Y2H
MORF2-Y2H-R	CCCCCTCGAGTCATCTTGTGTTTTCTC	Y2H
MORF9-Y2H-F	CCTTGAATTCGCTGCGACGGTGGATTC	Y2H
MORF9-Y2H-R	CCCCGGATCCTTAAGAGGAATCAGAGG	Y2H
MORF8-Y2H-F	CCTTGAATTCGGCGGCCTTGTGTCTGTCAAAGG	Y2H
MORF8-Y2H-R	CCTTGGATCCTTAACCCTGGTAGGGGTTGCCAC	Y2H
MORF3-Y2H-F	CTTCGAATTCTCCTCTTCCCGAACCAGCCTGGG	Y2H
MORF3-Y2H-R	CCCCGGATCCTTAAGCACTGGGCTTGTTGAACT	Y2H
MORF6-Y2H-F	CTTCGAATTCACCCGGATGGATAGGTCTGGCGG	Y2H
MORF6-Y2H-R	CTTCGGATCCTCAACGCATGTTCTCCCTCCGTC	Y2H
ECD1 FLAG-F	CCTTCCCGGGATGGCGATGATAAGCTTTTCGTT	Transgene
ECD1-FLAG-R	CCCGAGCTCTCACTTATCGTCGTCATCCTTGTA ATCCTTATCGTCGTCATCCTTGTAATCCTTATCG TCGTCATCCTTGTAATCCCAATAATCATTAC	Transgene
ECD1-GUS-F	CCTTTGGATCCTAGAACACACACCCTCC	GUS
ECD1-GUS-R	CCCCCCATGGACGATCCGTTACTGTTA	GUS
ECD1-RNAi-F	GATTCGGTTCTCTACAATTCGTT	RNAi
ECD1-RNAi-R	ATCAAAGTCCAAGTAACAACATT	RNAi
rps14-149	GAAAAUUCAUGGAAAAUUACAAUCCCCACCGCG	EMSA
rps14-80	AAAUAUCAUUUGAUUCGUCGAUCCUCAAAAAAG	EMSA
ECD1-MBP-F	CTTTGAGCTCCCATCATCATCATCATCATGAAAACC TGTATTTTCAGGGCCTCATGGCTCGTGATGGGATTC GTC	Protein expression
ECD1-MBP-R	CCCCGCGGCCGCTCACCAATAATCATTACAAGAAC AT	Protein expression
psbA-F	ATGACTGCAATTTTAGAGAGACGCG	RNA blot
psbA-R	TTATCCATTTGTAGATGGAGCCTCA	RNA blot

rbcL-F	TGTCACCACAAACAGAGACTAAAGC	RNA blot
rbcL-R	TCTACTCTTGGCCATCTAATTTATC	RNA blot
clpP-F	ATGCCTATTGGCGTTCCAAAAGTAC	RNA blot
clpP-R	TTATTGAACCGCTACAAGATCAACA	RNA blot
rpoA-F	ATGGTTCGAGAGAAAGTCAAAGTAT	RNA blot
rpoA-R	CTATTTTTTTTCTAGAATGTCTAAT	RNA blot
16S-F	AACGGCTGCTAATACCCCGTAGGCT	Probe a
16S-R	TCCAGTCACTAGCCCTGCCTTCGGC	Probe a
23S-F	AGGAGAGCACTCATCTTGGGGTGGG	Probe b
23S-R	TTCAAACGAGGAAAGGCTTACGGTG	Probe b
4.5S-F	GAAGGTCACGGCGAGACGAGCCGTT	Probe c
4.5S-R	GTTCAAGTCTACCGGTCTGTTAGGA	Probe c
5S-F	TATTCTGGTGTCCTAGGCGTAGAGG	Probe d
5S-R	ATCCTGGCGTCGAGCTATTTTTCCG	Probe d
16S 5’-F	AACCCAATGAATGTGAGTTTTTC	Probe e
16S 5’-R	TTCATAGTTGCATTACTTATAGC	Probe e
16S 3’-F	TCAGGGAGAGCTAATGCTTCTTG	Probe f
16S 3’-R	GTCGTGCGGGCCTCCTGCTGGGG	Probe f
23S 3’-F	ATTCCGACTTCCCCAGAGCCTCC	Probe g
23S 3’-R	TCTTGAATTCTCAAAACTTCTGT	Probe g
4.5S 3’-F	CTTGTTCCTACATGACCTGATCA	Probe h
4.5S 3’-R	TCGAACCATGAACGAAGAAAGGC	Probe h
trnI-F	GGGCTATTAGCTCAGTGGTAGAG	Probe i
trnI-R	TGGGCCATCCTGGATTTGAACCA	Probe i
trnA-F	GGGGATATAGCTCAGTTGGTAGA	Probe j
trnA-R	TGGAGATAAGCGGACTCGAACCG	Probe j
